# Porous Honeycomb Self-Assembled Monolayers: Tripodal Adsorption and Hidden Chirality of Carboxylate Anchored Triptycenes on Ag

**DOI:** 10.1021/acsnano.1c03626

**Published:** 2021-06-14

**Authors:** Saunak Das, Giulia Nascimbeni, Rodrigo Ortiz de la Morena, Fumitaka Ishiwari, Yoshiaki Shoji, Takanori Fukushima, Manfred Buck, Egbert Zojer, Michael Zharnikov

**Affiliations:** † Angewandte Physikalische Chemie, Universität Heidelberg, Im Neuenheimer Feld 253, D-69120 Heidelberg, Germany; ‡ Institute of Solid State Physics, NAWI Graz, Graz University of Technology, Petersgasse 16, 8010 Graz, Austria; § EaStCHEM School of Chemistry, University of St Andrews, North Haugh, St Andrews KY16 9ST, U.K.; ∥ Laboratory for Chemistry and Life Science, Institute of Innovative Research, Tokyo Institute of Technology, 4259 Nagatsuta, Midori-ku, Yokohama 226-8503, Japan

**Keywords:** self-assembled monolayers, triptycene, polymorphism, chirality, scanning tunneling microscopy, density functional theory calculations

## Abstract

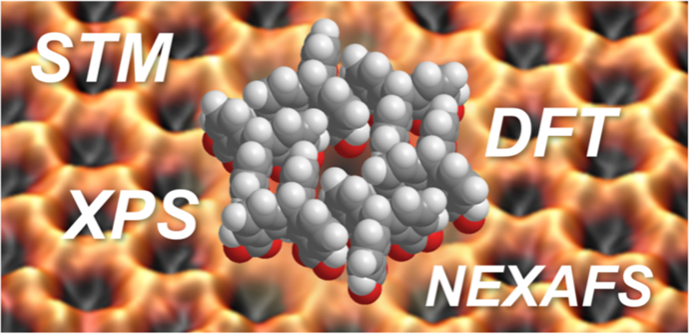

Molecules with tripodal anchoring to substrates represent a versatile platform for the fabrication of robust self-assembled monolayers (SAMs), complementing the conventional monopodal approach. In this context, we studied the adsorption of 1,8,13-tricarboxytriptycene (Trip-CA) on Ag(111), mimicked by a bilayer of silver atoms underpotentially deposited on Au. While tripodal SAMs frequently suffer from poor structural quality and inhomogeneous bonding configurations, the triptycene scaffold featuring three carboxylic acid anchoring groups yields highly crystalline SAM structures. A pronounced polymorphism is observed, with the formation of distinctly different structures depending on preparation conditions. Besides hexagonal molecular arrangements, the occurrence of a honeycomb structure is particularly intriguing as such an open structure is unusual for SAMs consisting of upright-standing molecules. Advanced spectroscopic tools reveal an equivalent bonding of all carboxylic acid anchoring groups. Notably, density functional theory calculations predict a chiral arrangement of the molecules in the honeycomb network, which, surprisingly, is not apparent in experimental scanning tunneling microscopy (STM) images. This seeming discrepancy between theory and experiment can be resolved by considering the details of the actual electronic structure of the adsorbate layer. The presented results represent an exemplary showcase for the intricacy of interpreting STM images of complex molecular films. They are also further evidence for the potential of triptycenes as basic building blocks for generating well-defined layers with unusual structural motifs.

## Introduction

Self-assembled monolayers (SAMs) are an important part of modern nanotechnology, with versatile applications ranging from corrosion protection and design of biointerfaces to lithography, nanofabrication, molecular electronics, organic electronics, and photovoltaics.^1−9^ SAMs usually consist of rodlike molecules featuring an anchoring group, mediating the bonding to a specific substrate, a (functional) tail group, constituting the SAM–ambient interface, and a backbone, connecting both groups and building the SAM matrix.^1−3^ Usually, each SAM-forming molecule comprises only a single docking group, but molecules with potentially dipodal,^10−12^ tripodal,^10,13−32^ and tetrapodal^32−34^ building configurations (bearing a suitable number of anchoring groups) have been designed as well. Such systems, in particular, target at a better electronic coupling to the substrate, a better control of molecular orientation, a reliable assembly of bulky moieties to highly organized layers, and a control of the density of functional tail groups and specific receptors. Among the aforementioned bonding configurations, the tripodal one is probably the most promising option, since a symmetric adsorption geometry offers a stable upright orientation of the functional tail groups. Accordingly, a variety of tripods, capable of building SAMs on various substrates, have been designed.^10,13−32^ In most cases, these tripods feature a tetrahedral core, defined by a central sp^3^-hybridized carbon^13,18,22,23,29^ or silicon atom,^14,16,17,20,21,24,26,31^ or a multiatom scaffold such as adamantane.^15,19,20,25,27^ This, in principle, allows the functionalization of the respective molecules with three anchoring groups and one functional tail group directed upright away from the substrate. Unfortunately, an actually tripodal adsorption configuration has rarely been achieved and, usually, only a fraction of the anchoring groups binds strongly to the substrate. The others either bind only weakly, remain unbound, or cross-link to neighbor molecules. As a result, ill-defined layers with low packing densities and limited lateral and orientational order are often formed. Such layers are then hardly capable of fulfilling the task they were initially designed for.

This prompted advanced molecular-design strategies to realize highly uniform and structurally well-defined SAMs with tripodal binding configuration, and one possible strategy builds on the triptycene (Trip) moiety, which is well-known for its ability to efficiently self-assemble.^35,36^ Thus, in an initial series of experiments, two triptycene based molecules were designed. Three thiol anchoring groups were attached to the 1,8,13-positions of the triptycene scaffold either directly (Trip-SH) or via a methylene linker (Trip-CH_2_-SH).^37^ The thiol groups were chosen in view of their affinity to coinage metals, such as Au, Ag, and Cu,^2^ and to semiconductor substrates, such as GaAs.^38−40^ On Au(111) (as the most popular support for SAMs)^2^ both molecules were found to form dense, nested hexagonal monolayers with large area structural uniformity. Most importantly, these layers feature a tripodal bonding configuration, yielding SAMs of high crystallinity in the Trip-CH_2_-SH case and resulting in a less perfect but still acceptable film quality in the case of Trip-SH.^37^ Similar considerations apply to Trip-CH_2_-SH on Ag(111) substrates.^41^

**Figure 1:**
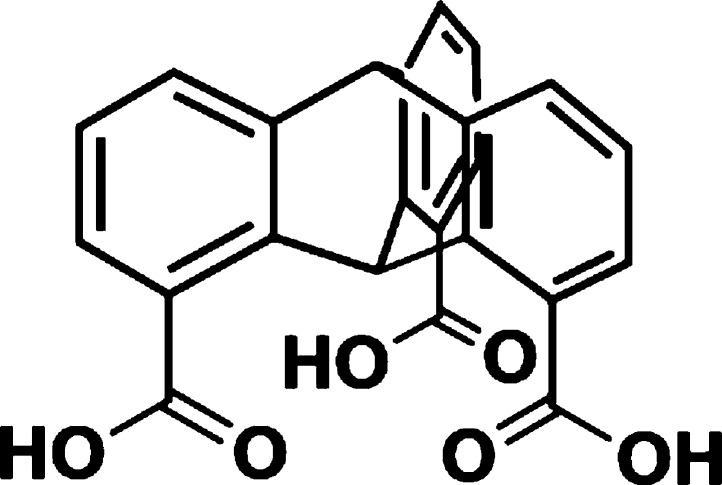
Molecular structure of Trip-CA.

Encouraged by the above successes in fabricating triptycene based tripodal monolayers, in the present study we take another important step to establish the general applicability of the triptycene approach by varying the docking groups. As a test molecule, we synthesized 1,8,13-tricarboxytriptycene (Trip-CA) featuring carboxylic acid (CA) anchoring groups attached to the 1,8,13-positions of the triptycene scaffold (Figure 1). CA is a reliable and suitable anchor for a variety of application-relevant substrates, including coinage metals such as Ag and Cu^42−45^ and metal oxides such as indium tin oxide and zinc oxide.^46,47^ Among these substrates we chose Ag(111) as a well-defined and application-relevant support, mimicking it with a bilayer of silver atoms underpotentially deposited on Au. Analogous substrates have been used by some of us for a variety of CA based SAM studies, exhibiting a reproducible quality and chemical character.^42,48,49^

For the characterization and study of the Trip-CA SAMs, we used a combination of complementary microscopic and spectroscopic techniques, *viz.* scanning tunneling microscopy (STM), X-ray photoelectron spectroscopy (XPS), and near-edge X-ray absorption fine structure (NEXAFS) spectroscopy, as well as density functional theory simulations providing insight into the molecular organization and electronic structure of these systems. It turned out that Trip-CA not only forms well-defined SAMs on Ag(111) but also displays a pronounced polymorphism. Of particular interest is a porous, honeycomb phase formed by the upright-standing triptycenes. That phase is also an instructive showcase illustrating that the obvious interpretation of STM images might be misleading.

## Results and Discussion

### STM Experiments

For the supramolecular assembly of sterically demanding molecules, polymorphism is often observed and the obtained structures are sensitively dependent on the preparation parameters.^50,51^ This also occurs in the case of Trip-CA SAMs for which in total four structures were observed. Note that the preparation temperature was kept at 363 K for all samples while the concentration of Trip-CA in the solution and the immersion time were varied (see Experimental Section for technical details).

**Figure 2:**
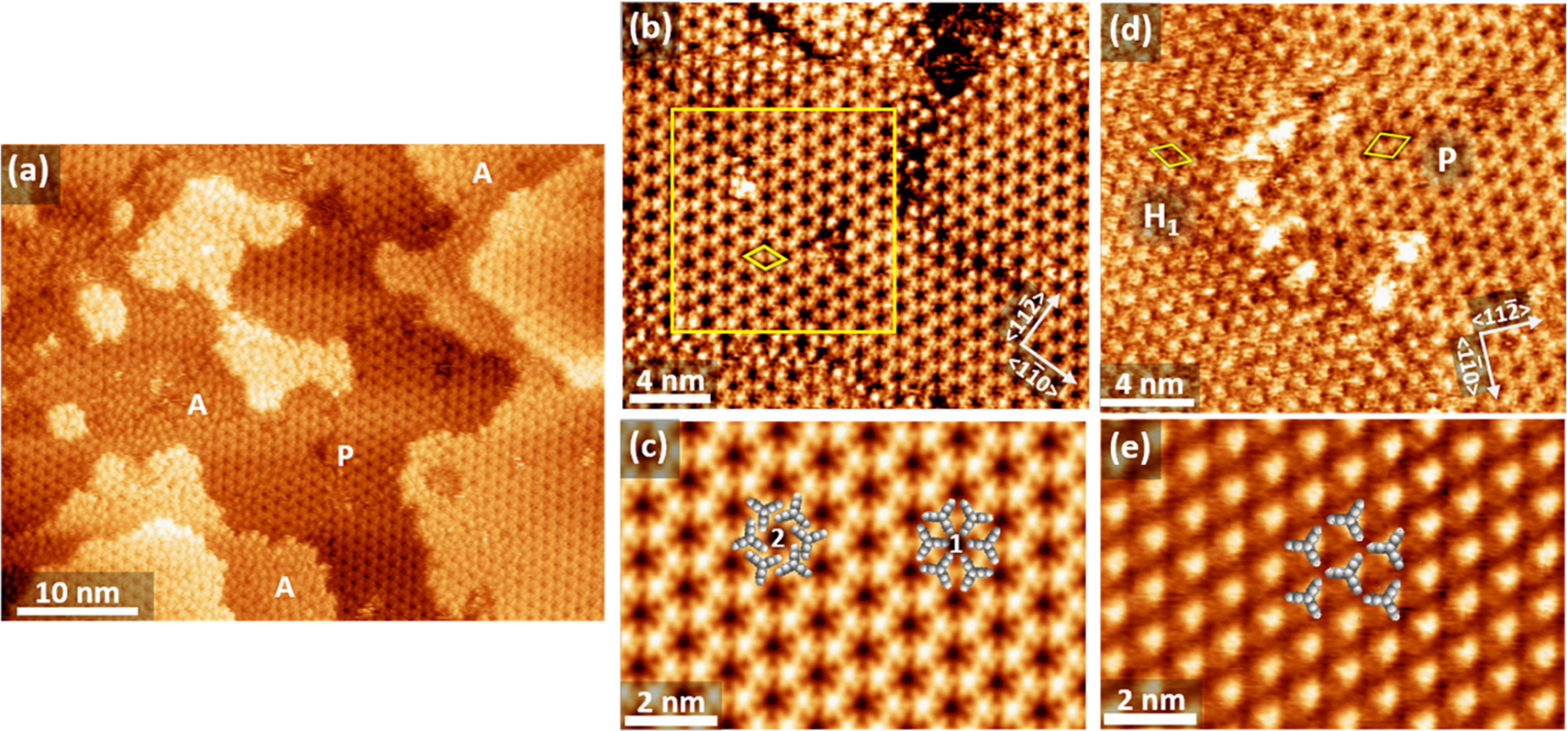
STM images of Trip-CA SAMs on UPD-Ag/Au/mica showing different phases. All samples were prepared at *T* = 363 K but at different concentrations of the solution and for different immersion times. (a) Large-scale image showing a porous, honeycomb-like structure (P) and disordered regions (A); preparation at 0.01 mM/30 min. (b) High resolution image of the honeycomb structure; preparation at 0.1 mM/30 min. (c) Unit cell averaged image of the area framed by the yellow square in part b together with two molecular models (1 and 2) detailed in the main text. (d) High resolution image showing adjacent domains of a hexagonally packed phase (H_1_) and the P-phase; preparation at 0.01 mM/30 min. The unit cells of the two phases are indicated by the yellow diamonds. (e) Unit cell averaged image of the H_1_-phase with the molecular model overlaid (from a different area of the sample shown in part d).

The dominant structure formed at short immersion times for both 0.01 mM and 0.1 mM solutions was an extended porous, honeycomb-like network, labeled P-phase. A representative image of this structure for the sample prepared by 30 min immersion into a 0.01 mM solution is shown in Figure 2a. Under these conditions the P-phase is accompanied by local areas of a disordered arrangement of molecules (A). The unit cell of the P-phase is a diamond aligned with the ⟨11̅0⟩ directions of the Ag(111) surface and a side length of 11.4 ± 0.5 Å. This alignment with the substrate lattice suggests a commensurate structure, and indeed, the derived length of the unit cell vectors is in full agreement with the 11.56 Å of a (4 × 4) structure. Further details of the porous structure are revealed by images as shown in Figure 2b which exhibit submolecular features. The most salient features are the distinct triangular shape of the protrusions and the links between them. This becomes even more clear in the unit cell averaged image displayed in Figure 2c. It seems obvious to identify the triangular shape with the contour of the upright-standing triptycene molecule, *i.e.*, associate the bright center of the triangle with the bridging carbon atom and align the corners of the triangle with the aromatic rings, as illustrated by model 1 overlaid with the STM image on the right half of Figure 2c. However, one has to keep in mind that a purely geometrical interpretation of STM images can be misleading due to the crucial influence of the electronic structure.^52−55^ Geometrically, a structure such as the one shown by model 2 on the left half is also conceivable, and in fact, it might be even preferred over the structure of model 1 as this arrangement suggests increased π–π and van der Waals interactions between molecules. We will return to this issue when presenting the calculations in the Computational Studies section and merely note at this point that achiral molecules which, like triptycenes, exhibit a 3-fold symmetry can assemble into chiral structures such as model 2.^56−59^

In addition to the porous structure and local amorphous areas, another ordered phase (labeled H_1_ in Figure 2d) is observed for the samples prepared at a concentration of 0.01 mM. It is characterized by a hexagonal packing with molecules separated by 10 Å. Its unit cell, which can be described by a (2√3 × 2√3) structure, is aligned with the ⟨112̅⟩ direction of the substrate. The respective area per molecule is 86.6 Å^2^, which is almost 50% larger than the 57.87 Å^2^ for the P-phase. Looking at the unit cell averaged image of Figure 2e, the molecular shape is not as distinct as in Figure 2c, but some triangular geometry is discernible. Tentatively aligning the molecules along the shape of the protrusions yields the structural model displayed in Figure 2e. Since the density of the H_1_-phase is much lower than that of the P-phase, the interaction between the molecules must be rather weak and, therefore, it can be expected that this phase is not stable. Indeed, this is observed in our experiments, as the H_1_-phase is not seen when the SAM is prepared at a concentration of 0.1 M or when the 0.01 mM solution is used, but immersion times are extended to a few hours.

**Figure 3:**
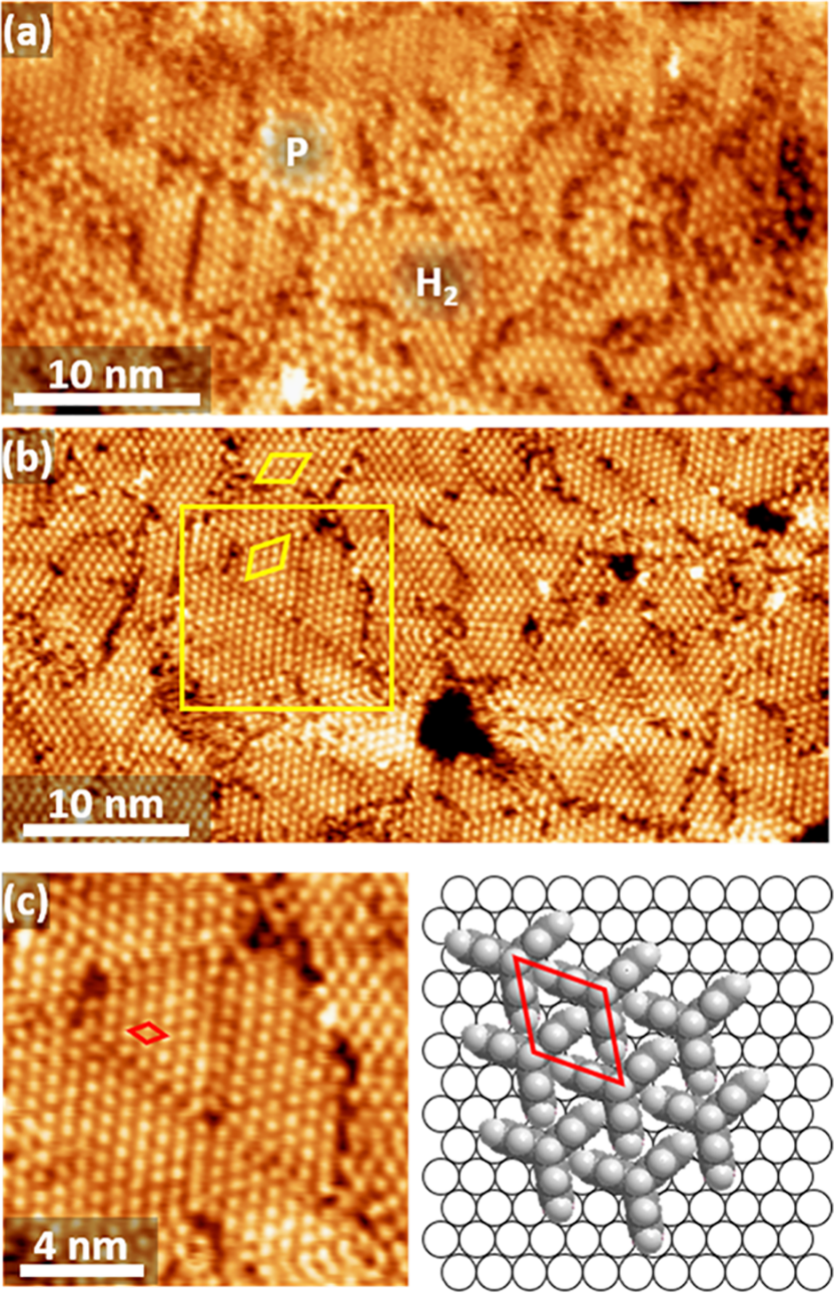
High resolution STM images of Trip-CA SAMs on UPD-Ag/Au/mica prepared at *T* = 363 K. (a) Sample prepared from 0.01 mM solution and immersed for 16 h showing islands of the honeycomb structure (P) and a hexagonally close-packed phase (H_2_). (b) Sample prepared from 0.1 mM solution and immersed for 16 h displaying only the H_2_ structure. Yellow diamonds indicate unit cells (multiple size shown for clarity) related by mirror symmetry. (c) Enlarged image of the section marked by the yellow square in part c and structural model of the H_2_ phase, with the respective (√7×√7)R19.1° unit cell represented by the red diamonds. The Ag surface is only shown to illustrate the orientation of the molecular lattice. The exact positions of the adsorption sites are not known.

Even though the honeycomb structure (P-phase) can be prepared in good yield, it is also metastable to some extent. For the 0.01 mM solution, small patches of yet another phase appear, when the Ag substrate is immersed for a few hours. For even longer times, formation of the emerging phase progresses until the P-phase is disrupted as illustrated in Figure 3a, where the SAM obtained after an adsorption time of 16 h is shown. Only small patches of the porous network structure remain, and the monolayer is dominated by another hexagonally packed phase, labeled H_2_. At the higher concentration of 0.1 M and the same immersion time of 16 h, the SAM only consists of the H_2_ phase as illustrated in Figure 3b. The structure of this phase can be accurately determined from a SAM where the H_2_ and P structures coexist, with the latter serving as reference. It turns out that the H_2_ structure is not aligned with the low index directions of the substrates but is off the ⟨11̅0⟩ direction by ±19°. Consequently, mirror domains are observed as indicated by the yellow diamonds in Figure 3b. With an experimentally determined intermolecular distance of 7.6 Å the structure is also commensurate and described by a (√7×√7)R19.1° unit cell. This gives an area per molecule of 50.63 Å^2^ (1.97 × 10^14^ molecules/cm^2^), which is 14% denser than the honeycomb structure. This requires the molecules to adopt the nested packing shown in Figure 3c and reported earlier for other triptycene based systems.^37,41,60^ However, the packing of the molecules in the Trip-CA SAMs on Ag is significantly denser than in the analogous thiol SAMs on Au(111) and Ag(111) (∼65 Å^2^ per molecule)^37,41^ and also denser than that reported for thin multilayer films of tripodal paraffinic triptycenes (56.8 Å^2^ per molecule).^60^

Another aspect worth mentioning is that, in contrast to the studies of the thiolate-bonded triptycene SAMs,^37,41^ for the P phase of the Trip-CA SAM the maximum (apparent) height is located in the center of the triangular shape, *i.e.*, at the bridging carbon atom, an aspect that will also be addressed below, when discussing the STM simulations. It is worth noting that the distinct triangular shape of the individual molecules is seen only for the porous phase. For the other phases the triangular shape is either rather faint (*e.g.*, Figure 2d,e) or not perceptible at all.

Briefly summarizing the STM studies, four phases were observed, *viz.* (i) a disordered structure, (ii) a low density hexagonally packed structure, unstable at higher concentrations and/or long adsorption times (H_1_-phase); (iii) a porous honeycomb-like structure (P-phase), and (iv) another hexagonal structure (H_2_-phase), which is significantly denser than the H_1_-phase and forms for long immersion times.

Among these phases, only H_2_ and P can be prepared reliably as majority structures under suitable experimental conditions. Even though the H_2_ phase is characterized by a particularly high packing density, it exhibits a “conventional” hexagonal molecular arrangement, similar to those reported already for Trip-SH and Trip-CH_2_-SH SAMs on Au(111) and Trip-CH_2_-SH SAM on Ag(111).^37,41^ In contrast, the honeycomb-like P-phase represents a structure distinctly different from all others that have so far been observed for triptycene based SAMs. Such open structures, frequently described as porous networks,^61^ are rather typical of molecules with flat adsorption geometry, such as trimesic acid,^62,63^ 1,3,5-benzenetribenzoic acid,^51^ or hexaazatriphenylene-hexanitrile^64^ on noble metal substrates. In the present case, we have a fundamentally different situation, namely a rather open structure formed by upright-standing and tightly anchored molecules, which makes this phase unusual and particularly interesting. Moreover, for the P-phase the STM images exhibit submolecular features, which enable a detailed investigation of how in such structures STM images correlate with the actual arrangement of the molecules. Consequently, in our further studies we will exclusively focus on the characterization and analysis of the P-phase.

### Spectroscopic Characterization

For the spectroscopic studies, samples of the P-phase of Trip-CA SAM on UPD-Ag/Au/mica were prepared from a 0.01 mM solution into which the substrate was immersed for 4 h. The P-phase character of these samples was verified by STM prior to delivery to the synchrotron.

**Figure 4:**
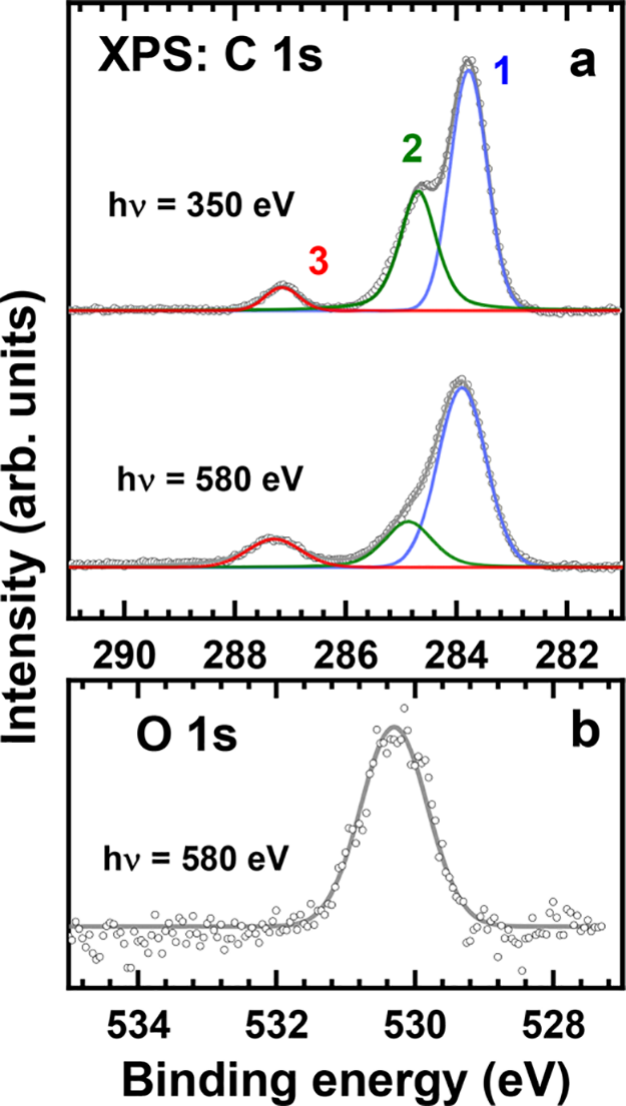
Background-corrected C 1s (a) and O 1s (b) XP spectra of the Trip-CA SAM on UPD-Ag/Au/mica (P-phase). The C 1s spectra correspond to photon energies of 350 and 580 eV; they are decomposed into individual peaks, drawn in different colors and marked by numbers. The O 1s spectrum was acquired at a photon energy of 580 eV and fitted by a single peak.

The C 1s and O 1s XP spectra of the Trip-CA SAM on UPD-Ag/Au/mica are shown in Figure 4. The C 1s spectra in Figure 4a correspond to two different photon energies and, consequently, to two different kinetic energies of photoelectrons, with a larger signal attenuation and, respectively, higher surface sensitivity for the 350 eV spectrum.^65^ These spectra are tentatively decomposed into three individual peaks marked by numbers, even though we cannot exclude that the joint feature 1 and 2 contains even more components hidden within the common envelope. Peaks 1 and 2 can be assigned to the triptycene unit and peak 3 to the carbon atoms in the docking CA groups. The BE of this peak, ∼287.2 eV matches the literature value for the carboxylate (COO^–^) group bound to silver^48,49^ but is distinctly different from that for the unbound COOH group (∼288.5 eV).^48,49^ Significantly, this peak is comparably narrow, corresponding to a well-defined chemical state of the involved carbon atoms. Consequently, we can conclude that all three docking groups of the Trip-CA molecules in the respective SAMs are bound to the substrate in the same bidentate fashion.

The assignment of the dominant peaks 1 and 2, associated with the triptycene scaffold and located at 283.85 and 284.8 eV, respectively, is not entirely straightforward but is assisted by the analysis of their spectral weights as a function of photon energy (PE), with the smaller PE (350 eV) typically amplifying contributions from atoms located at and close to the SAM–ambient interface. While the relative spectral weight of peak 1 varies only slightly upon PE variation (59.3% at 350 eV and 64.8% at 580 eV), that of the peak 2 changes significantly, decreasing from 34.3% to 18.8% when increasing the PE from 350 to 580 eV (the signal of the carboxylate groups changes, as expected, in the opposite fashion, from 6.4% to 16.4%). Consequently, the carbon atoms corresponding to peak 2 should be located at the top of the adsorbed molecules. The intensity of this peak suggests that it should be related to several carbon atoms per molecule. Unfortunately, the exact identification of these atoms has not been possible even with the help of our simulations, as these, unfortunately, do not provide full, quantitative agreement with the experiments in the case of triptycene monolayers, as discussed already in ref (37).

As to peak 1, its energy (283.85 eV) is somewhat lower than the analogous value for the SAMs of thiolate-anchored triptycenes on Au(111) (284.1 eV)^37^ and that for SAMs of monodentate-anchored oligophenylenecarboxylic acids on Ag(111) (284.0–284.1 eV).^48^ This suggests that the C 1s XP spectra of the Trip-CA SAMs are affected to some extent by electrostatic effects,^66^ associated, most likely, with the dipoles of the polar anchoring groups bonded to the substrate.

The O 1s XP spectrum in Figure 4b exhibits just one, well-defined peak at ∼530.3 eV, assigned to the oxygen atoms in the anchoring groups. Its energetic position agrees exactly with that for the anchoring carboxylate groups in a variety of CA SAMs on Ag (530.3–530.4 eV), and it is distinctly different from the energies associated with the carbonyl (∼531.4 eV) and hydroxyl (∼533 eV) signals of unbound −COOH moieties.^48,49^ Thus, in agreement with the C 1s data, all oxygen atoms in the anchoring groups of the Trip-CA molecules are in the same chemical state, corresponding to a bidentate bonding of the carboxylate groups to the substrate.

Complementary information is provided by the NEXAFS data, which, for the sake of brevity, are presented in the Supporting Information. The spectra of the Trip-CA exhibit characteristic absorption resonances of the Trip-CA molecules with no evidence for any contamination. A quantitative analysis of the spectra yields an average tilt angle of ∼9° between the axis of the Trip-CA and the surface normal. This value is quite close to the analogous parameter for the Trip-CH_2_-SH SAM on Au(111) (∼7.5°) also exhibiting a tripodal bonding to the substrate, mediated by the thiolate anchoring groups connected to the triptycene framework via methylene linkers.^37^ The deviation from the fully upright orientation of the Trip-CA molecules in the SAM could be, on the one hand, explained by a possible corrugation of the specific anchoring sites of individual carboxylate groups. This is, however, not observed in the simulations (see below). An alternative explanation would be the presence of small regions of the disordered amorphous phase (see STM Experiments section) as well as the occurrence of defects, such as domain boundaries or step edges, distorting the arrangement of the Trip-CA molecules within the P-phase.

### Computational Studies

**Figure 5:**
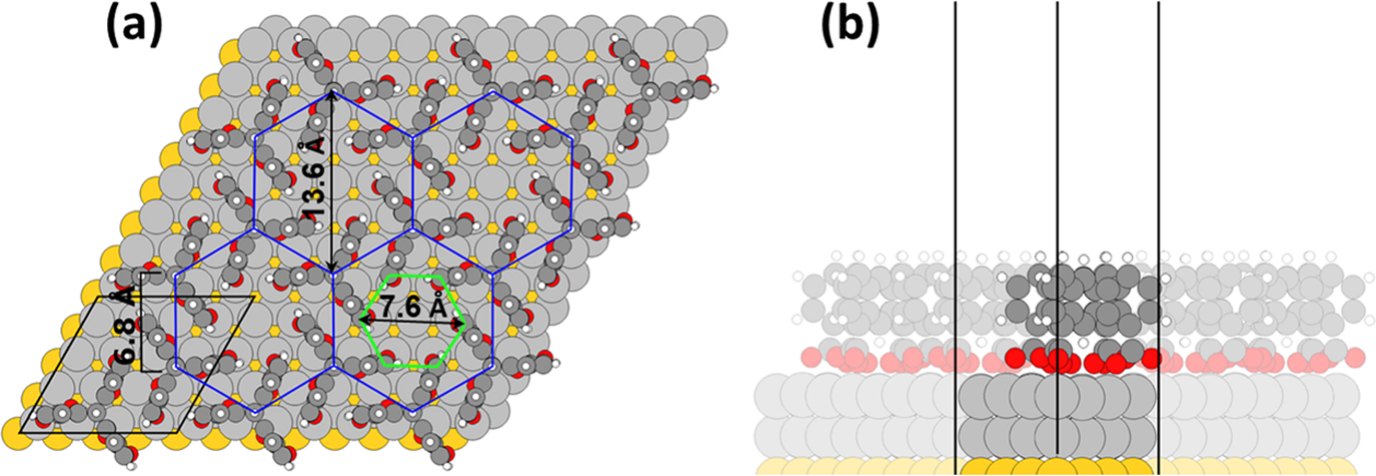
Top (a) and side (b) views of the optimized Trip-CA/Ag(111)/Au(111) geometry in the hexagonal phase. Au atoms are depicted in dark yellow, Ag atoms in light gray, O atoms in red, C atoms in dark gray, and H atoms in white. The black lines enclose the unit cell.

A key task of the computations was to establish which structure the molecules in the honeycomb P-phase actually adopt (see structure models “1” and “2” in Figure 2c). The final outcome of an extensive series of simulations of two Trip-CA molecules adsorbed in a 4 × 4 unit cell on two layers of Ag on a Au(111) substrate is shown in Figure 5. On the surface, the molecules assemble into a honeycomb pattern comprised of hexagons with a side-length of 6.8 Å and a diameter of 13.6 Å (see blue hexagons in Figure 5). The size of the pores is rather small, with a diameter of the pore-hexagon of 7.6 Å (green hexagon in Figure 5, defined by the innermost carbon atoms of the triptycenes), which is ∼4.5 times the van der Waals radius of a carbon atom.

This structure clearly matches model “2” from Figure 2 with a chiral arrangement of the molecules. This is surprising, considering that the STM images of the P-phase in Figure 2 do not contain any indication for a significant rotation of the molecules (where it should be mentioned that clockwise and counterclockwise rotations would be isoenergetic). Rather, in the experiments the triangular contours of the molecules point toward the center of the hexagonal pores.

Therefore, to test whether the molecular arrangement shown in Figure 5 is just a local minimum geometry, we considered several alternative starting geometries comprising rotated Trip-CA molecules as well as rotated docking groups.^67^ They, however, all ended up in the same structure with a chiral arrangement of the molecules and with the two carboxylic oxygen atoms in each linker located close to on-top and bridge positions of the substrate. This raises the question whether a nonchiral arrangement of the molecules could be caused by the presence of metal (Ag) adatoms on the surface. To test that, we considered five different periodic surface reconstruction motifs comprising between one and six adatoms per unit cell. The chosen starting geometries as well as the optimized structures are shown in the Supporting Information. Significantly, in none of the investigated cases did the presence of the adatoms prevent the formation of a chiral structure.

**Figure 6:**
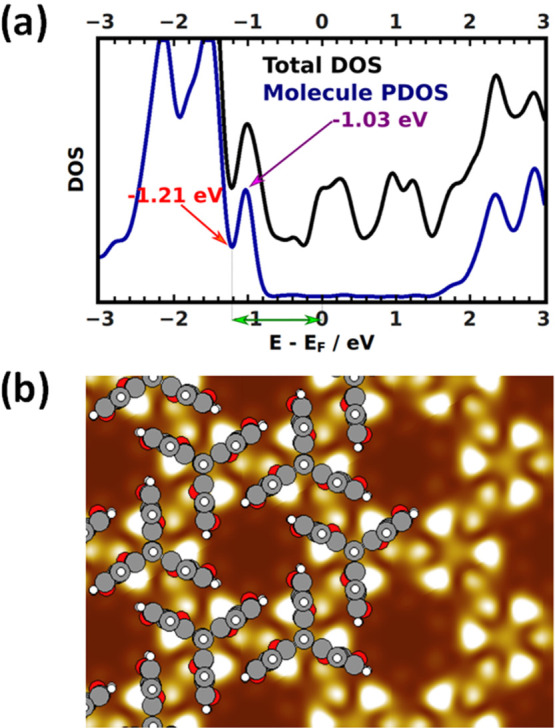
(a) Total density of states (DOS; black line) and DOS projected on the molecule (PDOS; blue line) for the porous structure of Trip-CA on Ag/Au. The purple arrow indicates the peak of the PDOS originating from the band derived from the molecular HOMO of Trip-CA, the red arrow indicates the energy of the PDOS minimum right below that peak, and the green double-sided arrow highlights the energy range considered in the STM simulations. (b) Cut through the local density of states (LDOS) integrated in a 0.1 eV energy window centered at the first peak of the molecular PDOS in panel (a) at −1.03 eV. The LDOS is plotted for a plane located 1.8 Å above the position of the center of the highest atom in the assembled triptycene molecules.

Considering that in all our simulations of the porous phase of Trip-CA on Ag/Au a chiral structure comes out as the most stable one, the question arises what could be the reason for not being able to resolve chirality in the STM experiments. To investigate that, it is necessary to go beyond the mere geometry of the system and also to consider its electronic structure. Bearing in mind that the experimental STM images have been obtained for a positive tip bias, we concentrated on occupied states at the interface and here in particular on the first peak that can be associated with a molecular feature, *i.e.*, the peak associated with the bands derived from the molecular HOMO (there are two such bands, as there are two molecules per unit cell). In the projected density of states (PDOS) in Figure 6a, the associated feature has a maximum at −1.03 eV. In passing we note that the calculated peak position for a variety of reasons (including the neglect of screening and the many-electron self-interaction error) does not necessarily coincide quantitatively with the position of the feature relevant for (resonant) tunneling in the STM experiments. Nevertheless, even if only the tail of the HOMO-derived bands was contained in the experimental bias voltage window, it would very likely still dominate the shape of the observed STM features. Thus, the HOMO-derived bands with the peak in the PDOS at −1.03 eV will serve as a reference for the following considerations.

The calculated local density of states (LDOS) for an energy window of 0.1 eV around that peak is shown in Figure 6b for a plane 1.8 Å above the center of the topmost atom in the assembled triptycene molecules. This figure reveals several aspects. First, the regions of maximum LDOS do not coincide with the blades of the triptycenes; rather, they are located on either side of these blades (*i.e.*, phenylene rings), which is not surprising considering that the highest-occupied electronic states in the triptycenes possess π-character. This is also consistent with the three-leaved clover type shape of isolated, thiolate-bonded triptycenes in the STM images by Chaunchaiyakul et al.^41^ Second, the brightness of the π-lobes on the two sides of each ring is not the same. A similar asymmetry of STM-related features has been observed, for example, for anthraceneselenolate SAMs, and this has been attributed to the molecular tilt.^68^ However, this explanation can be ruled out here, considering the only marginal calculated tilt of the molecules relative to the surface normal (see the Supporting Information). In fact, in the present case, the asymmetry arises from the relative arrangement of neighboring Trip-CA molecules, where one side of each molecular blade faces an essentially parallel blade of a neighboring molecule, while the other side of the blade faces the edge of a blade of another adjacent molecule.

**Figure 7:**
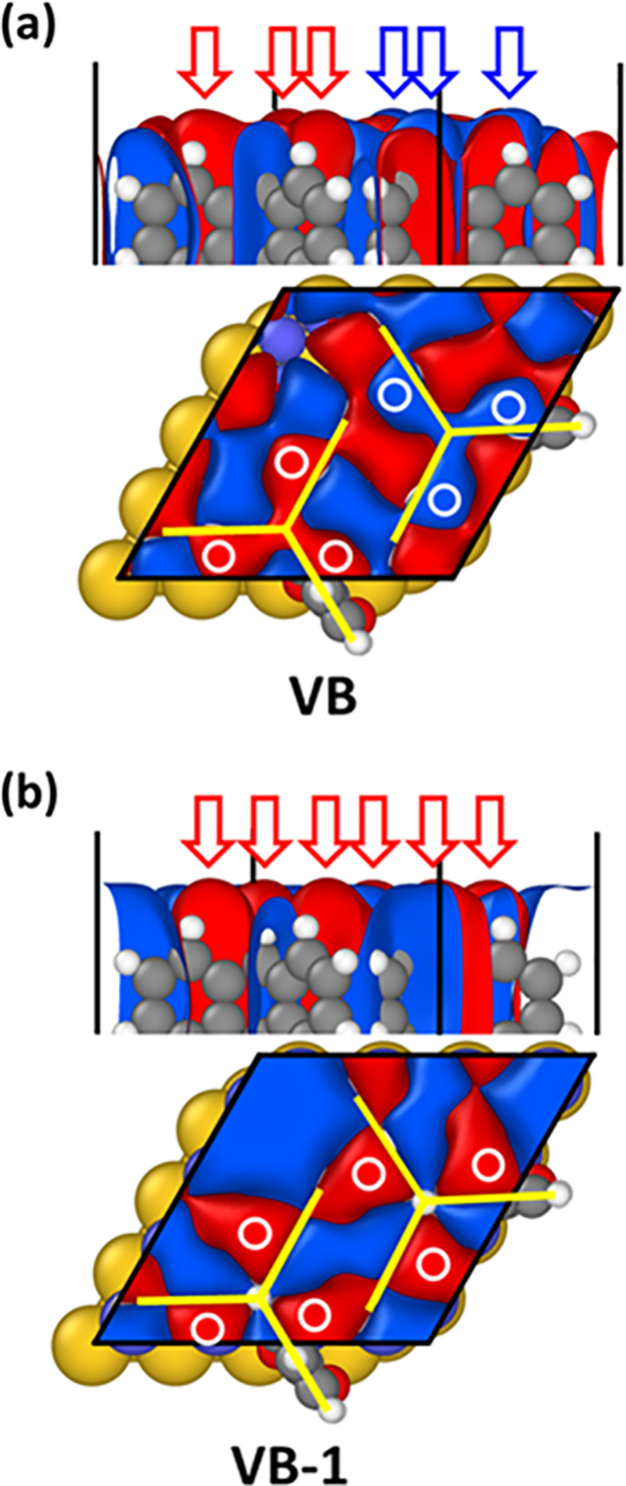
Isodensity plot of the 2829th (panel b) and 2830th (panel a) Γ-point eigenstate of the Trip-CA SAM on Ag/Au calculated for the 4 × 4 surface unit cell. These states correspond to the two highest occupied bands primarily associated with the adsorbate layer and derived from the molecular HOMOs. The states are located 0.97 eV (VB) and 1.04 eV (VB-1) below the Fermi level of the interface. The red and blue arrows as well as the white circles denote the orbital lobes protruding furthest into the vacuum above the interface. The black lines indicate the unit cells, and the yellow lines denote the backbones of the triptycene molecules. To better visualize the spreading of the orbitals into the vacuum above the interface, a very small isovalue (0.0007 Å^3/2^) has been chosen for plotting the electronic states.

For the two bands derived from the triptycene HOMOs, this results in the Γ-point Kohn–Sham states shown in Figure 7, which are located at 0.97 eV below the Fermi level for the “molecular valence band”, VB, and at 1.04 eV for the VB-1. The regions, in which the orbitals from neighboring molecules form bonding or antibonding linear combinations, differ for the two states. Nevertheless, a side-view of the electronic states in the top panels of Figures 7a and 7b reveals that the lobes of the wave functions that protrude somewhat further into the vacuum (highlighted by arrows and circles) are the same in both bands. Notably, these lobes are always on the same sides of the blades of the triptycenes, fully consistent with the LDOS plotted in Figure 6b.

Interestingly, already the LDOS in Figure 6b indicates that, when considering the actual electronic structure of the adsorbate layer, the chiral arrangement of the molecules is no longer clearly resolved. This means that the absence of a chiral structure in the experiments is not the consequence of such a structure not being formed. Rather, it is due to the specific shape of the electronic states in the monolayer and the way in which they determine the STM image.

A notable difference between the experimental STM image of the porous phase in Figure 2c and the calculated LDOS around the HOMO-derived peak in Figure 6b is that the bright feature in the center of the molecule that is observed in the experiments is not visible in the simulations. This discrepancy is, however, resolved as soon as one no longer calculates the LDOS only around the HOMO-derived peak but rather integrates over all occupied states between the Fermi level and the entire HOMO-derived peak (*i.e.*, to −1.21 eV – see red arrow in Figure 6a). The resulting graphs, which (within the Tersoff–Hamann approach) correspond to constant height STM images for an infinitely sharp tip, are shown in the Supporting Information for different tip heights.

**Figure 8:**
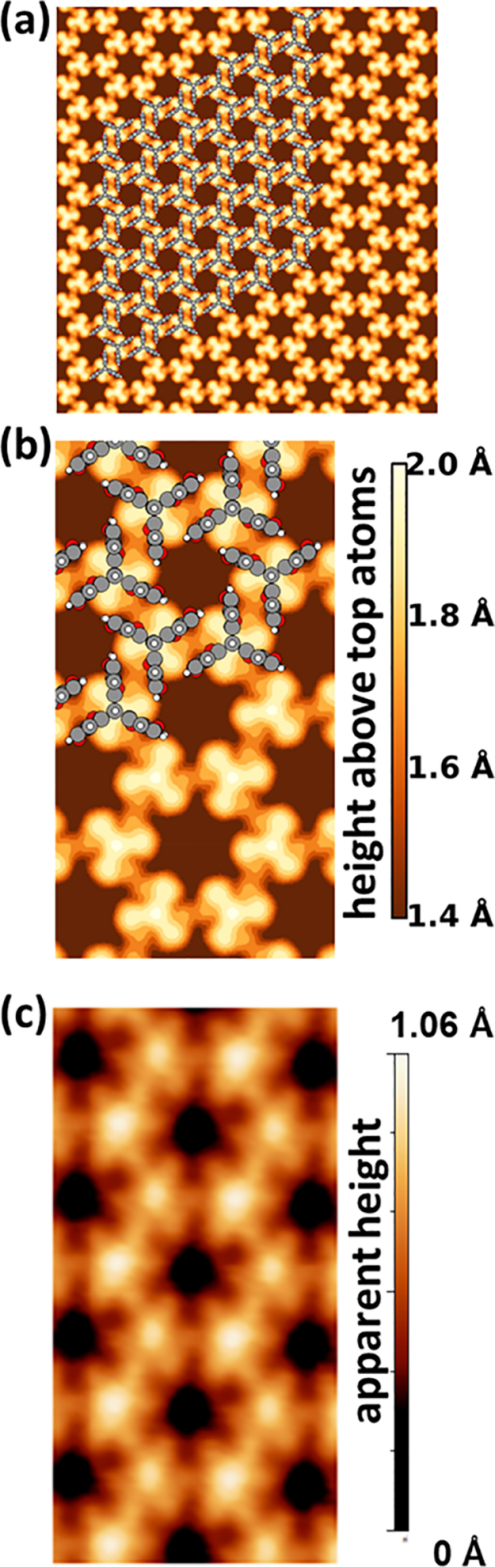
(a) Calculated constant-current STM image for the Trip-CA monolayer on the Ag/Au(111) substrate calculated for a positive bias of 1.21 V and assuming a spherical tip with a radius of 1 Å. The structure of the monolayer as obtained in the geometry optimization process of the interface is superimposed on part of the calculated image. (b) Zoom into panel a. (c) Zoom into the experimental image shown in Figure 2c.

As a final step, to allow for a direct comparison between simulations and experiments, we calculated constant current STM images “blurred” by assuming a tip with a finite extent (radius of 1 Å) and integrating between the Fermi level and −1.21 eV. The corresponding calculated images are shown in Figures 8a and 8b (partly with an overlaid structure of the molecules). For ease of comparison, panel c contains a zoom into the experimental image from Figure 2c. From the data shown in Figure 8 one sees that (i) there is an excellent agreement between the simulated and measured STM images, (ii) the molecules adopt a chiral structure within the elementary cells, which is just “hidden” in the experiment, as there one does not resolve the geometrical but the electronic structure of the adsorbate layer, and (iii) the bright spots seen in the STM do not correspond to the blades of the molecules but can rather be associated with peculiar features of the π-electron system of a Trip-CA monolayer adopting the porous phase on a Ag/Au substrate.

## Conclusions

Self-assembly of Trip-CA on Ag(111) was studied by a combination of STM, X-ray spectroscopies and computational methods. According to the STM data, several different monolayer structures can be formed, depending on preparation conditions such as solution concentration and immersion time. Along with frequently detected hexagonal molecular arrangements, a porous honeycomb-like structure is observed. Such a structure is not unusual for molecules adopting a flat adsorption geometry,^64,69−71^ but it is rather unexpected for SAMs comprised of upright-standing molecules. The resulting corrugated interfacial patterns could be attractive for confining nanoscopic objects on a surface in a well-ordered pattern despite the rather small diameter of the pores.

According to the spectroscopic data, all molecules in this structure are adsorbed in a well-defined tripodal geometry, with all three carboxylate docking groups bonded to the substrate as bidentate. This adsorption geometry is characterized by an essentially upright orientation of the benzene rings forming the “blades” of the Trip-CA molecules. Extensive theoretical simulations reveal the internal organization of the porous structure with the intriguing outcome that the molecules arrange in a chiral fashion, an aspect that is not resolved in the STM images despite their submolecular resolution. This apparent conundrum can be resolved by showing that the most pronounced features seen in the STM images do not represent the geometric arrangement of the molecules but are rather a consequence of the peculiar shapes of the orbitals resulting from the hybridization of the highest occupied π-states of neighboring molecules. In fact, the simulations show that, considering the finite extent of the experimental tip, achiral STM images are to be expected, despite the chiral arrangement of the molecules.

Regarding SAM design, our results underpin the potential of the triptycene scaffold as a platform for tripodal molecular assembly. Considering that carboxylic acids represent suitable docking groups for a variety of application-relevant substrates beyond coinage metals, such as indium tin oxide and zinc oxide,^46,47,72^ there is significant potential of molecules derived from the Trip-CA motive. In contrast to Trip-CH_2_-SH, where the docking group is separated from the triptycene by an “insulating” CH_2_ spacer,^37,41^ here the π-electron system of the CA group is in the immediate vicinity of the triptycene. Moreover, the π-planes of the CA entities and the triptycene blades nearly coincide (see the structure in Figure 5a). This can be expected to promote a good electronic coupling between the substrate and the triptycenes (including potential functional substituents), an aspect that can be highly advantageous for a variety of applications.

## Experimental Section

### Synthesis

**Scheme 1:**
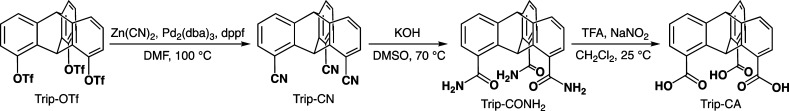
Synthesis of Trip-CA

The synthesis procedure is illustrated in Scheme 1. 1,8,13-Tris(trifluoromethanesulfonyloxy)triptycene (Trip-OTf) was prepared according to previously reported procedures.^37^ Nuclear magnetic resonance (NMR) spectroscopy measurements were carried out on a Bruker model AVANCE III HD-500 spectrometer (^1^H: 500.0 MHz, ^13^C: 125.7 MHz). Chemical shifts (δ) are expressed relative to the resonances of the residual nondeuterated solvent for ^1^H (CD_3_CN: ^1^H(δ) = 1.94 ppm, DMSO-*d*_6_: ^1^H(δ) = 2.05 ppm) and the resonances of the residual solvent for ^13^C (CD_3_CN: ^13^C(δ) = 118.26, 1.32 ppm, DMSO: ^13^C(δ) = 39.52 ppm). Absolute values of the coupling constants are given in Hertz (Hz), regardless of their sign. Multiplicities are abbreviated as singlet (s), doublet (d), triplet (t), quartet (q), multiplet (m), and broad (br). Infrared (IR) spectra were recorded at 25 °C on a JASCO model FT/IR-660*_Plus_* Fourier transform IR spectrometer. Mass spectrometry measurements were carried out on a Bruker model micro TOF II mass spectrometer, equipped with an atmospheric pressure chemical ionization (APCI) probe.

#### 1,8,13-Tricyanotriptycene (Trip-CN)

Under argon, Trip-OTf (1.56 g, 2.23 mmol) was added to a dry DMF solution (22 mL) of a mixture of 1,1′-bis(diphenylphosphino)ferrocene (dppf, 1.22 g, 2.22 mmol), tris(dibenzylideneacetone)dipalladium (Pd_2_(dba)_3_, 0.60 g, 1.35 mmol), and Zn(CN)_2_ (2.31 g, 19.7 mmol) at 25 °C, and the resulting mixture was stirred at 100 °C for 12 h. After being allowed to cool to room temperature, the reaction mixture was poured into an aqueous solution of NH_3_ (28%, 50 mL), and the precipitate formed was collected by filtration, washed successively with aqueous ammonia, water, and CH_2_Cl_2_, and then dried under reduced pressure. The residue was subjected to Soxhlet extraction with acetone, and the extract was evaporated to dryness under reduced pressure. The resultant residue was washed with cold acetone and then evaporated to dryness under reduced pressure to afford Trip-CN as a colorless powder (630 mg) in 86% yield: FT-IR (KBr): ν (cm^–1^) 3425, 3025, 2673, 2561, 2229, 1693, 1427, 1300, 1207, 1170, 804, 766, 713. ^1^H NMR (500 MHz, CD_3_CN): δ (ppm) 7.91 (d, *J* = 7.6 Hz, 3H), 7.56 (d, *J* = 7.8 Hz, 3H), 7.37 (dd, *J* = 7.8, 7.6 Hz, 3H), 6.74 (s, 1H), 6.23 (s, 1H). ^13^C NMR (125 MHz, CD_3_CN): δ (ppm) 148.5, 144.2, 134.2, 128.9, 124.8, 119.0, 52.5, 37.3. APCI-TOF mass: calcd. for C_23_H_11_N_3_ [M]^+^: *m*/*z* = 329.0947; found: 329.0948.

#### 1,8,13-Tris(aminocarbonyl)triptycene (Trip-CONH_2_)

A DMSO solution (76 mL) of a mixture of Trip-CN (625 mg, 1.90 mmol) and KOH (5.36 g, 95.1 mmol) was stirred at 70 °C for 24 h and cooled to 0 °C. An aqueous solution of HCl (1 M, 95 mL) was slowly added to the resulting mixture, and the precipitate formed was collected by filtration, washed with water, and dried under reduced pressure to give Trip-CONH_2_ as a pale-yellow powder (485 mg) in 67% yield: FT-IR (KBr): ν (cm^–1^) 3193, 1650, 1612, 1427, 1401, 768, 634. ^1^H NMR (500 MHz, DMSO-*d*_6_): δ (ppm) 8.18 (s, 3H), 7.56 (d, *J* = 7.5 Hz, 3H), 7.55 (s, 3H), 7.41 (s, 1H), 7.20 (d, *J* = 7.4 Hz, 3H), 7.07 (dd, *J* = 7.5, 7.4 Hz, 3H), 5.80 (s, 1H). ^13^C NMR (125 MHz, DMSO-*d*_6_): δ (ppm) 169.5, 146.5, 141.8, 133.2, 125.3, 125.0, 124.7, 52.8, 43.3. APCI-TOF mass: calcd. for C_23_H_17_N_3_O_3_ [M]^+^: *m*/*z* = 383.1264; found: 383.1262.

#### 1,8,13-Tricarboxytriptycene (Trip-CA)

NaNO_2_ (2.4 g, 34.5 mmol) was added to a CH_2_Cl_2_ solution (13 mL) of a mixture of Trip-CONH_2_ (204 mg, 0.532 mmol) and trifluoroacetic acid (13 mL, 34.5 mmol) at 0 °C, and the mixture was stirred at 25 °C for 12 h. The precipitate formed was collected by filtration, washed with water, and dried under reduced pressure. The residue was recrystallized from acetone to give Trip-CA as a colorless solid (185 mg) in 90% yield: FT-IR (KBr): ν (cm^–1^) 3073, 2670, 2553, 1693, 1596, 1431, 1305, 1169, 775, 661. ^1^H NMR (500 MHz, DMSO-*d*_6_): δ (ppm) 8.35 (s, 1H), 7.63 (d, *J* = 7.3 Hz, 3H), 7.40 (d, *J* = 7.7 Hz, 3H), 7.11 (dd, *J* = 7.7, 7.3 Hz, 3H), 5.78 (s, 1H). ^13^C NMR (125 MHz, DMSO-*d*_6_): δ (ppm) 167.9, 147.0, 144.3, 129.4, 126.7, 125.9, 125.1, 52.6, 43.5. APCI-TOF mass: calcd. for C_23_H_14_O_6_ [M]^+^: *m*/*z* = 386.0785; found: 386.0782.

### Substrates

Silver substrates were mimicked by a bilayer of silver atoms on Au. The Au supports were purchased from Georg Albert PVD, Silz, Germany, as a 300 nm epitaxial Au(111) layer on mica slides. Cut to size, these supports were annealed using a natural gas flame followed by underpotential (UPD) deposition of Ag. In the course of the deposition process, Au/mica was immersed in 10 mM AgNO_3_ in 100 mM HNO_3_ (aq) and a potential of 10 mV (vs Ag/Ag^+^) was applied for 2 min. As the result, a full coverage, pseudomorphic (1 × 1) Ag bilayer was formed, adopting the well-defined, (111)-terminated surface of the Au support.^73^

### Preparation of SAMs

For SAM fabrication, the UPD Ag/Au/mica substrates were immersed in an aqueous solution of Trip-CA. Concentrations of 0.1 and 0.01 M were employed, and immersion times varied from 30 min to 16 h. The preparation temperature was 90 °C in all cases. The parameters of the immersion procedure were selected on the basis of our experience regarding the preparation of CA-anchored SAMs and optimized to some extent in a series of preliminary experiments.

### SAM Characterization

The SAMs were characterized by scanning tunneling microscopy (STM), X-ray photoelectron spectroscopy (XPS), and near edge X-ray absorption fine structure (NEXAFS) spectroscopy. All measurements were performed at room temperature. The spectroscopic experiments were conducted in ultrahigh vacuum, at a base pressure of *ca.* 1 × 10^–9^ mbar.

SAMs were imaged in ambient environment with a Molecular Imaging PicoSTM, controlled by Picoscan software V5.3.3. Tips were manually cut from a 0.25 mm diameter Pt/Ir wire (80:20, hard-tempered, Advent Research Material Ltd.). Images were recorded in constant current mode with tunneling currents in the range of 1–100 pA. The tip bias was in the range of 0.200–0.800 V and positive as imaging was more stable using this polarity. Images were evaluated using WSxM software.^74^

XPS and NEXAFS experiments were performed at the bending magnet HE-SGM beamline of the synchrotron storage ring BESSY II in Berlin, Germany, using a custom-designed experimental station.^75^ The XP spectra were measured with a Scienta R3000 electron energy analyzer, in normal emission geometry. The primary photon energy (PE) was set to either 350 or 580 eV to access specific core levels and to vary the surface sensitivity. The energy resolution at these PEs was ∼0.3 eV and ∼0.5 eV, respectively. The binding energy (BE) scale of the spectra was referenced to the Au 4f_7/2_ emission of the Au substrate at 84.0 eV.^76^ When necessary for their analysis, the spectra were fitted by symmetric Voigt functions and a linear background.

The NEXAFS spectra were collected at the carbon and oxygen K-edges in the partial electron yield (PEY) mode with retarding voltages of −150 V and −350 V, respectively. As the primary X-ray source, linearly polarized synchrotron light with a polarization factor of ∼89% was used. The incidence angle of the X-rays was varied between the normal (90°) and grazing (20°) incidence geometry to monitor the linear dichroism reflecting the molecular orientation in the SAMs.^77^ The energy resolution was ∼0.3 eV at the C K-edge and ∼0.5 eV at the O K-edge. The PE scale was referenced to the pronounced π* resonance of HOPG at 285.38 eV.^78^ The relative shift of the O K-edge range was estimated using reference XPS measurements. The raw spectra were corrected for the PE dependence of the incident photon flux and reduced to the standard form with zero intensity in the pre-edge region and the unity jump in the far postedge region.

### Computational Methods

Calculations were performed employing the FHI-aims code^79−82^ in conjunction with the PBE functional^83^ and the surface version^84^ of the Tkatchenko–Scheffler van der Waals correction^85^ (explicitly ignoring van der Waals interactions between metal atoms). Periodic boundary conditions and the so-called repeated slab approach were used to describe extended surfaces. For the final calculations of the best suited adsorbate structure of the porous phase (see below), a 5 × 5 × 1 k-point mesh in combination with a 4 × 4 surface unit cell containing two molecules was employed (see the Computational Studies section). We used FHI-aims specific “tight” basis functions in combination with the default numerical settings with the exception of Ag. For this species a more extended cutoff potential was used to better represent the electronic structure of the metal surface. Further details on the used basis functions can be found in the Supporting Information. To determine the occupation of the Kohn–Sham eigenstates, we used a Gaussian broadening function with a width of σ = 0.1 eV. The metal substrate was modeled by three layers of Au (which were kept fixed during the geometry optimizations to mimic the bulk structure) and two layers of Ag (in analogy to the experimental situation). The positions of the Ag atoms and all atoms in the adsorbate layer were fully relaxed. We also performed an extensive screening of possible alternative structures using (i) different starting geometries and (ii) considering a variety of adatom structures. For these calculations, less stringent settings were used, including “light” basis sets and only three metal layers (one layer of Au and two layers of Ag). For the calculations on the final structure (the screening calculations) the total energy criterion for the self-consistency cycle was set to 10^–6^ (10^–5^) eV and the geometries were optimized until the maximum residual force component per atom was below 0.01 (0.05) eV/Å. The dimensions of the unit cells in the x and y directions are given in units of the theoretically calculated bulk nearest neighbor distance of 2.94 Å corresponding to a lattice constant for the fcc cell of 4.158 Å. The motivation for employing the theoretical nearest neighbor distance rather than the experimental value of 2.89 Å is that in this way spurious surface relaxations in the geometry optimization process can be avoided. The total height of the unit cell in the direction perpendicular to the surface was set to 40 Å, generating a vacuum region of at least 20 Å to quantum-mechanically decouple the periodic replicas of the slab in that direction. To decouple them also electrostatically, a self-consistently calculated dipole correction was used.^86^

STM images were simulated within the Tersoff–Hamann approximation^87^ following the procedure described in detail in ref (88). The FHI-aims generated STM cube files were postprocessed using a routine written by Dr. Oliver T. Hofmann, from the Institute of Solid State Physics of the Graz University of Technology. This routine approximates constant-current images by an isosurface of the local DOS integrated between the Fermi level and the tip bias. To account for the finite extent of the tip, the local DOS is averaged over points arranged on the surface of a spherical tip with a tip radius of 1.0 Å.^88^ The considered bias voltage is discussed in the Computational Studies section. The aforementioned procedure allows generation of constant current as well as constant height images.

Isodensity plots of eigenstates were produced using OVITO;^89^ otherwise, the graphical user interface of the Atomic Simulation Environment (ASE)^90^ was used to generate molecular structure. The generated STM images were plotted using XCrysDen^91^ and matplotlib.^92^

## Supporting Information Available

NEXAFS spectroscopy data and their analysis; computational details; additional computational data (PDF)

## References

[ref1] Ulman A. Formation and Structure of Self-Assembled Monolayers. Chem. Rev. 1996, 96, 1533–1554. 10.1021/cr9502357. 11848802

[ref2] Love J. C.; Estroff L. A.; Kriebel J. K.; Nuzzo R. G.; Whitesides G. M. Self-Assembled Monolayers of Thiolates on Metals as a Form of Nanotechnology. Chem. Rev. 2005, 105, 1103–1169. 10.1021/cr0300789. 15826011

[ref3] Eck W. Chemisorption of Polyatomic Chain-Like Hydrocarbons on Metals and Semiconductors. In Landolt-Börnstein - Group III Condensed Matter; Bonzel H. P., Ed.; Springer-Verlag: Berlin, Heidelberg, 2005; Vol. 42A4.

[ref4] Halik M.; Hirsch A. The Potential of Molecular Self-Assembled Monolayers in Organic Electronic Devices. Adv. Mater. 2011, 23, 2689–2695. 10.1002/adma.201100337. 21823250

[ref5] Turchanin A.; Gölzhäuser A. Carbon Nanomembranes. Adv. Mater. 2016, 28, 6075–6103. 10.1002/adma.201506058. 27281234

[ref6] Vilan A.; Cahen D. Chemical Modification of Semiconductor Surfaces for Molecular Electronics. Chem. Rev. 2017, 117, 4624–4666. 10.1021/acs.chemrev.6b00746. 28230354

[ref7] Sizov A. S.; Agina E. V.; Ponomarenko S. A. Self-Assembled Semiconducting Monolayers in Organic Electronics. Russ. Chem. Rev. 2018, 87, 1226–1264. 10.1070/RCR4839.

[ref8] Telegdi J. Formation of Self-Assembled Anticorrosion Films on Different Metals. Materials 2020, 13, 5089 10.3390/ma13225089. PMC7697528 33187283

[ref9] Terfort A.; Zharnikov M. Electron-Irradiation Promoted Exchange Reaction as a Tool for Surface Engineering and Chemical Lithography. Adv. Mater. Interfaces 2021, 8, 2100148 10.1002/admi.202100148.

[ref10] Chinwangso P.; Jamison A. C.; Lee T. R. Multidentate Adsorbates for Self-Assembled Monolayer Films. Acc. Chem. Res. 2011, 44, 511–519. 10.1021/ar200020s. 21612198

[ref11] Ford W. E.; Gao D.; Knorr N.; Wirtz R.; Scholz F.; Karipidou Z.; Ogasawara K.; Rosselli S.; Rodin V.; Nelles G.; von Wrochem F. Organic Dipole Layers for Ultralow Work Function Electrodes. ACS Nano 2014, 8, 9173–9180. 10.1021/nn502794z. 25093963

[ref12] Rittikulsittichai S.; Park C. S.; Jamison A. C.; Rodriguez D.; Zenasni O.; Lee T. R. Bidentate Aromatic Thiols on Gold: New Insight Regarding the Influence of Branching on the Structure, Packing, Wetting, and Stability of Self-Assembled Monolayers on Gold Surfaces. Langmuir 2017, 33, 4396–4406. 10.1021/acs.langmuir.7b00088. 28383920

[ref13] Hirayama D.; Takimiya K.; Aso Y.; Otsubo T.; Hasobe T.; Yamada H.; Imahori H.; Fukuzumi S.; Sakata Y. Large Photocurrent Generation of Gold Electrodes Modified with [60]Fullerene-Linked Oligothiophenes Bearing a Tripodal Rigid Anchor. J. Am. Chem. Soc. 2002, 124, 532–533. 10.1021/ja016703d. 11804479

[ref14] Jian H.; Tour J. M. En Route to Surface-Bound Electric Field-Driven Molecular Motors. J. Org. Chem. 2003, 68, 5091–5103. 10.1021/jo034169h. 12816462

[ref15] Kitagawa T.; Idomoto Y.; Matsubara H.; Hobara D.; Kakiuchi T.; Okazaki T.; Komatsu K. Rigid Molecular Tripod with an Adamantane Framework and Thiol Legs. Synthesis and Observation of an Ordered Monolayer on Au(111). J. Org. Chem. 2006, 71, 1362–1369. 10.1021/jo051863j. 16468783

[ref16] Weidner T.; Krämer A.; Bruhn C.; Zharnikov M.; Shaporenko A.; Siemeling U.; Träger F. Novel Tripod Ligands for Prickly Self-Assembled Monolayers. Dalton Trans. 2006, 23, 2767–2777. 10.1039/B515727G. 16751884

[ref17] Shirai Y.; Cheng L.; Cheng B.; Tour J. M. Characterization of Self-Assembled Monolayers of Fullerene Derivatives on Gold Surfaces: Implications for Device Evaluations. J. Am. Chem. Soc. 2006, 128, 13479–13489. 10.1021/ja063451d. 17031961

[ref18] Nikitin K.; Lestini E.; Lazzari M.; Altobello S.; Fitzmaurice D. A Tripodal [2]Rotaxane on the Surface of Gold. Langmuir 2007, 23, 12147–12153. 10.1021/la701657r. 17963409

[ref19] Katano S.; Kim Y.; Matsubara H.; Kitagawa T.; Kawai M. Hierarchical Chiral Framework Based on a Rigid Adamantane Tripod on Au(111). J. Am. Chem. Soc. 2007, 129, 2511–2515. 10.1021/ja065893v. 17279745

[ref20] Weidner T.; Zharnikov M.; Hoβbach J.; Castner D. G.; Siemeling U. Adamantane-Based Tripodal Thioether Ligands Functionalized with a Redox-Active Ferrocenyl Moiety for Self-Assembled Monolayers. J. Phys. Chem. C 2010, 114, 14975–14982. 10.1021/jp104376p. PMC3049913 21399702

[ref21] Ramachandra S.; Schuermann K. C.; Edafe F.; Belser P.; Nijhuis C. A.; Reus W. F.; Whitesides G. M.; De Cola L. Luminescent Ruthenium Tripod Complexes: Properties in Solution and on Conductive Surfaces. Inorg. Chem. 2011, 50, 1581–1591. 10.1021/ic1002868. 21194229

[ref22] Zhu S.-E; Kuang Y.-M.; Geng F.; Zhu J.-Z.; Wang C.-Z.; Yu Y.-J.; Luo Y.; Xiao Y.; Liu K.-Q.; Meng Q.-S.; Zhang L.; Jiang S.; Zhang Y.; Wang G.-W.; Dong Z.-C.; Hou J. G. Self-Decoupled Porphyrin with a Tripodal Anchor for Molecular-Scale Electroluminescence. J. Am. Chem. Soc. 2013, 135, 15794–15800. 10.1021/ja4048569. 24066644

[ref23] Sakamato R.; Ohirabaru Y.; Matsuoka R.; Maeda H.; Katagiri S.; Nishihara H. Orthogonal Bis(terpyridine)-Fe(II) Metal Complex Oligomer Wires on a Tripodal Scaffold: Rapid Electron Transport. Chem. Commun. 2013, 49, 7108–7110. 10.1039/c3cc42478b. 23615896

[ref24] Chen K.-Y.; Ivashenko O.; Carroll G. T.; Robertus J.; Kistemaker J. C. M.; London G.; Browne W. R.; Rudolf P.; Feringa B. L. Control of Surface Wettability Using Tripodal Light-Activated Molecular Motors. J. Am. Chem. Soc. 2014, 136, 3219–3224. 10.1021/ja412110t. 24490770

[ref25] Kitagawa T.; Matsubara H.; Komatsu K.; Hirai K.; Okazaki T.; Hase T. Ideal Redox Behavior of the High-Density Self-Assembled Monolayer of a Molecular Tripod on a Au(111) Surface with a Terminal Ferrocene Group. Langmuir 2013, 29, 4275–4282. 10.1021/la305092g. 23470152

[ref26] Chen K.-Y.; Ivashenko O.; Carroll G. T.; Robertus J.; Kistemaker J. C. M.; London G.; Browne W. R.; Rudolf P.; Feringa B. L. Control of Surface Wettability Using Tripodal Light-Activated Molecular Motors. J. Am. Chem. Soc. 2014, 136, 3219–3224. 10.1021/ja412110t. 24490770

[ref27] Kitagawa T.; Matsubara H.; Okazaki T.; Komatsu K. Electrochemistry of the Self-Assembled Monolayers of Dyads Consisting of Tripod-Shaped Trithiol and Bithiophene on Gold. Molecules 2014, 19, 15298–15313. 10.3390/molecules190915298. 25255246 PMC6271350

[ref28] Valášek M.; Lindner M.; Mayor M. Rigid Multipodal Platforms for Metal Surfaces. Beilstein J. Nanotechnol. 2016, 7, 374–405. 10.3762/bjnano.7.34. 27335731 PMC4901557

[ref29] Lindner M.; Valášek M.; Homberg J.; Edelmann K.; Gerhard L.; Wulfhekel W.; Fuhr O.; Wächter T.; Zharnikov M.; Kolivoška V.; Pospíšil L.; Mészáros G.; Hromadová M.; Mayor M. Importance of the Anchor Group Position (Para *versus* Meta) in Tetraphenylmethane Tripods: Synthesis and Self-Assembly Features. Chem. - Eur. J. 2016, 22, 13218–13235. 10.1002/chem.201602019. 27505302

[ref30] Valášek M.; Mayor M. Spatial and Lateral Control of Functionality by Rigid Molecular Platforms. Chem. - Eur. J. 2017, 23, 13538–13548. 10.1002/chem.201703349. 28766790

[ref31] Sánchez-Molina M.; Díaz A.; Sauter E.; Zharnikov M.; López-Romero J. M. Synthesis of Novel Tripod-Shaped Molecules and Their Immobilization on Au(111) Substrates. Appl. Surf. Sci. 2019, 470, 259–268. 10.1016/j.apsusc.2018.11.013.

[ref32] Li Z.-Q.; Tang J.-H.; Zhong Y.-W. Multidentate Anchors for Surface Functionalization. Chem. - Asian J. 2019, 14, 3119–3126. 10.1002/asia.201900989. 31389657

[ref33] Benneckendorf F. S.; Rohnacher V.; Sauter E.; Hillebrandt S.; Münch M.; Wang C.; Casalini S.; Ihrig K.; Beck S.; Jänsch D.; Freudenberg J.; Jaegermann W.; Samorì P.; Pucci A.; Bunz U. H. F.; Zharnikov M.; Müllen K. A Tetrapodal Diazatriptycene Enforces Orthogonal Orientation in Self-Assembled Monolayers. ACS Appl. Mater. Interfaces 2020, 12, 6565–6572. 10.1021/acsami.9b16062. 31825591

[ref34] Rohnacher V.; Benneckendorf F. S.; Münch M.; Sauter E.; Asyuda A.; Barf M.-M.; Tisserant J.-N.; Hillebrandt S.; Rominger F.; Jänsch D.; Freudenberg J.; Kowalsky W.; Jaegermann W.; Bunz U.; Pucci A.; Zharnikov M.; Müllen K. Functionalized Tetrapodal Diazatriptycenes for Electrostatic Dipole Engineering in *n*-Type Organic Thin Film Transistors. Adv. Mater. Technol. 2021, 6, 2000300 10.1002/admt.202000300.

[ref35] Liu J.; Wachter T.; Irmler A.; Weidler P. G.; Gliemann H.; Pauly F.; Mugnaini V.; Zharnikov M.; Wöll C. Electric Transport Properties of Surface-Anchored Metal-Organic Frameworks and the Effect of Ferrocene Loading. ACS Appl. Mater. Interfaces 2015, 7, 9824–9830. 10.1021/acsami.5b01792. 25875419

[ref36] Liu J.; Kind M.; Schüpbach B.; Käfer D.; Winkler S.; Zhang W.; Terfort A.; Wöll C. Triptycene-Terminated Thiolate and Selenolate Monolayers on Au(111). Beilstein J. Nanotechnol. 2017, 8, 892–905. 10.3762/bjnano.8.91. 28503400 PMC5405688

[ref37] Ishiwari F.; Nascimbeni G.; Sauter E.; Tago H.; Shoji Y.; Fujii S.; Kiguchi M.; Tada T.; Zharnikov M.; Zojer E.; Fukushima T. Triptycene Tripods for the Formation of Highly Uniform and Densely Packed Self-Assembled Monolayers with Controlled Molecular Orientation. J. Am. Chem. Soc. 2019, 141, 5995–6005. 10.1021/jacs.9b00950. 30869881 PMC6483319

[ref38] McGuiness C. L.; Shaporenko A.; Mars C. K.; Uppili S.; Zharnikov M.; Allara D. L. Molecular Self-Assembly at Bare Semiconductor Surfaces: Preparation and Characterization of Highly Organized Octadecanethiolate Monolayers on GaAs(001). J. Am. Chem. Soc. 2006, 128, 5231–5243. 10.1021/ja058657d. 16608359

[ref39] McGuiness C. L.; Shaporenko A.; Zharnikov M.; Walker A. V.; Allara D. L. Molecular Self-Assembly at Bare Semiconductor Surfaces: Investigation of the Chemical and Electronic Properties of the Alkanethiolate-GaAs(001) Interface. J. Phys. Chem. C 2007, 111, 4226–4234. 10.1021/jp065173a.

[ref40] McGuiness C. L.; Diehl G. A.; Blasini D.; Smilgies D. M.; Zhu M.; Samarth N.; Weidner T.; Ballav N.; Zharnikov M.; Allara D. L. Molecular Self-Assembly at Bare Semiconductor Surfaces: Cooperative Substrate Molecule Effects in Octadecanethiolate Monolayer Assemblies on GaAs(111), (110), and (100). ACS Nano 2010, 4, 3447–3465. 10.1021/nn1004638. 20481546

[ref41] Chaunchaiyakul S.; Zhang C.; Imada H.; Kazuma E.; Ishiwari F.; Shoji Y.; Fukushima T.; Kim Y. Self-Assembly Growth of an Upright Molecular Precursor with a Rigid Framework. J. Phys. Chem. C 2019, 123, 31272–31278. 10.1021/acs.jpcc.9b09948.

[ref42] Cebula I.; Lu H.; Zharnikov M.; Buck M. Monolayers of Trimesic and Isophthalic Acid on Cu and Ag: The Influence of Coordination Strength on Adsorption Geometry. Chem. Sci. 2013, 4, 4455–4464. 10.1039/c3sc52137k.

[ref43] Aitchison H.; Lu H.; Zharnikov M.; Buck M. Monolayers of Biphenyl-3,4’,5-Tricarboxylic Acid Formed on Cu and Ag from Solution. J. Phys. Chem. C 2015, 119, 14114–14125. 10.1021/acs.jpcc.5b01176.

[ref44] Krzykawska A.; Ossowski J.; Zaba T.; Cyganik P. Binding Groups for Highly Ordered SAM Formation: Carboxylic *versus* Thiol. Chem. Commun. 2017, 53, 5748–5751. 10.1039/C7CC01939D. 28492691

[ref45] Aitchison H.; Lu H.; de la Morena R. O.; Cebula I.; Zharnikov M.; Buck M. Self-Assembly of 1,3,5-Benzenetribenzoic Acid on Ag and Cu at the Liquid/Solid Interface. Phys. Chem. Chem. Phys. 2018, 20, 2731–2740. 10.1039/C7CP06160A. 29322147

[ref46] Yip H.-L.; Hau S. K.; Baek N. S.; Ma H.; Jen A. K.-Y. Polymer Solar Cells That Use Self-Assembled-Monolayer-Modified ZnO/Metals as Cathodes. Adv. Mater. 2008, 20, 2376–2382. 10.1002/adma.200703050.

[ref47] An D.; Liu H.; Wang S.; Li X. Modification of ITO Anodes with Self-Assembled Monolayers for Enhancing Hole Injection in OLEDs. Appl. Phys. Lett. 2019, 114, 153301 10.1063/1.5086800.

[ref48] Aitchison H.; Lu H.; Hogan S. W. L.; Fruchtl H.; Cebula I.; Zharnikov M.; Buck M. Self-Assembled Monolayers of Oligophenylenecarboxylic Acids on Silver Formed at the Liquid-Solid Interface. Langmuir 2016, 32, 9397–9409. 10.1021/acs.langmuir.6b01773. 27588836

[ref49] de la Morena R. O.; Asyuda A.; Lu H.; Aitchison H.; Turner K.; Francis S. M.; Zharnikov M.; Buck M. Shape Controlled Assembly of Carboxylic Acids: Formation of a Binary Monolayer by Intercalation into Molecular Nanotunnels. Phys. Chem. Chem. Phys. 2020, 22, 4205–4215. 10.1039/C9CP06724H. 32043099

[ref50] Suresh S. M.; Duda E.; Hall D.; Yao Z.; Bagnich S.; Slawin A. M. Z.; Bässler H.; Beljonne D.; Buck M.; Olivier Y.; Köhler A.; Zysman-Colman E. A Deep Blue B,N-Doped Heptacene Emitter That Shows Both Thermally Activated Delayed Fluorescence and Delayed Fluorescence by Triplet–Triplet Annihilation. J. Am. Chem. Soc. 2020, 142, 6588–6599. 10.1021/jacs.9b13704. 32134259

[ref51] Kampschulte L.; Lackinger M.; Maier A. K.; Kishore R. S. K.; Griessl S.; Schmittel M.; Heckl W. M. Solvent Induced Polymorphism in Supramolecular 1,3,5-Benzenetribenzoic Acid Monolayers. J. Phys. Chem. B 2006, 110, 10829–10836. 10.1021/jp057553m. 16771333

[ref52] Weiss P. S.; Eigler D. M. Site Dependence of the Apparent Shape of a Molecule in Scanning Tunneling Micoscope Images: Benzene on Pt {111}. Phys. Rev. Lett. 1993, 71, 3139–3142. 10.1103/PhysRevLett.71.3139. 10054867

[ref53] Lei S.; De Feyter S. STM, STS and Bias-Dependent Imaging on Organic Monolayers at the Solid–Liquid Interface. Top. Curr. Chem. 2008, 285, 269–312. 10.1007/128_2007_23. 23636680

[ref54] Shen C.; Buck M. Patterning of Self-Assembled Monolayers Based on Differences in Molecular Conductance. Nanotechnology 2009, 20, 245306–245306. 10.1088/0957-4484/20/24/245306. 19468158

[ref55] Katano S.; Kim Y.; Kitagawa T.; Kawai M. Tailoring Electronic States of a Single Molecule Using Adamantane-Based Molecular Tripods. Phys. Chem. Chem. Phys. 2013, 15, 14229–14233. 10.1039/c3cp51612a. 23877197

[ref56] Perdigão L. M. A.; Perkins E. W.; Ma J.; Staniec P. A.; Rogers B. L.; Champness N. R.; Beton P. H. Bimolecular Networks and Supramolecular Traps on Au (111). J. Phys. Chem. B 2006, 110, 12539–12542. 10.1021/jp060062x. 16800583

[ref57] Kühne D.; Klappenberger F.; Decker R.; Schlickum U.; Brune H.; Klyatskaya S.; Ruben M.; Barth J. V. Self-Assembly of Nanoporous Chiral Networks with Varying Symmetry from Sexiphenyl-Dicarbonitrile on Ag(111). J. Phys. Chem. C 2009, 113, 17851–17859. 10.1021/jp9041217.

[ref58] Mu Z.; Shu L.; Fuchs H.; Mayor M.; Chi L. Two Dimensional Chiral Networks Emerging from the Aryl–F···H Hydrogen-Bond-Driven Self-Assembly of Partially Fluorinated Rigid Molecular Structures. J. Am. Chem. Soc. 2008, 130, 10840–10841. 10.1021/ja801925q. 18651733

[ref59] Szabelski P.; Rzysko W.; Panczyk T.; Ghijsens E.; Tahara K.; Tobe Y.; De Feyter S. Self-Assembly of Molecular Tripods in Two Dimensions: Structure and Thermodynamics from Computer Simulations. RSC Adv. 2013, 3, 25159–25165. 10.1039/c3ra45342a.

[ref60] Seiki N.; Shoji Y.; Kajitani T.; Ishiwari F.; Kosaka A.; Hikima T.; Takata M.; Someya T.; Fukushima T. Rational Synthesis of Organic Thin Films with Exceptional Long-Range Structural Integrity. Science 2015, 348, 1122–1126. 10.1126/science.aab1391. 26045433

[ref61] Cicoira F.; Santato C.; Rosei F. Two-Dimensional Nanotemplates as Surface Cues for the Controlled Assembly of Organic Molecules. In STM and AFM Studies on (Bio)molecular Systems; Samori P., Ed.; Springer-Verlag: Berlin Heidelberg, 2008; Vol. 285, pp 203–268. 10.1007/128_2008_2 23636679

[ref62] Lackinger M.; Griessl S.; Markert T.; Jamitzky F.; Heckl W. M. Self-Assembly of Benzene–Dicarboxylic Acid Isomers at the Liquid Solid Interface: Steric Aspects of Hydrogen Bonding. J. Phys. Chem. B 2004, 108, 13652–13655. 10.1021/jp048248o.

[ref63] Ye Y.; Sun W.; Wang Y.; Shao X.; Xu X.; Cheng F.; Li J.; Wu K. A Unified Model: Self-Assembly of Trimesic Acid on Gold. J. Phys. Chem. C 2007, 111, 10138–10141. 10.1021/jp072726o.

[ref64] Glowatzki H.; Bröker B.; Blum R.-P.; Hofmann O. T.; Vollmer A.; Rieger R.; Müllen K.; Zojer E.; Rabe J. P.; Koch N. Soft” Metallic Contact to Isolated C_60_ Molecules. Nano Lett. 2008, 8, 3825–3829. 10.1021/nl8021797. 18954123

[ref65] Ratner M.; Castner D. Electron Spectroscopy for Chemical Analysis. In Surface Analysis - The Principal Techniques; Vickerman J., Ed.; Wiley: Chichester, 1997; pp 43–98.

[ref66] Taucher T. C.; Hehn I.; Hofmann O. T.; Zharnikov M.; Zojer E. Understanding Chemical *versus* Electrostatic Shifts in X-Ray Photoelectron Spectra of Organic Self-Assembled Monolayers. J. Phys. Chem. C 2016, 120, 3428–3437. 10.1021/acs.jpcc.5b12387. PMC4761973 26937264

[ref67] Nascimbeni G. Quantum Mechanical Simulations of Inorganic/Organic Hybrid Systems. PhD Thesis, Graz University of Technology, Graz, Austria, 2019.

[ref68] Track A. M.; Rissner F.; Heimel G.; Romaner L.; Käfer D.; Bashir A.; Rangger G. M.; Hofmann O. T.; Bučko T.; Witte G.; Zojer E. Simultaneously Understanding the Geometric and Electronic Structure of Anthraceneselenolate on Au(111): A Combined Theoretical and Experimental Study. J. Phys. Chem. C 2010, 114, 2677–2684. 10.1021/jp9102756.

[ref69] Madueno R.; Raisanen M. T.; Silien C.; Buck M. Functionalizing Hydrogen-Bonded Surface Networks with Self-Assembled Monolayers. Nature 2008, 454, 618–621. 10.1038/nature07096. 18668104

[ref70] Li Z.; Han B.; Wan L. J.; Wandlowski T. Supramolecular Nanostructures of 1,3,5-Benzene-Tricarboxylic Acid at Electrified Au(111)/0.05 M H_2_SO_4_ Interfaces: An *in Situ* Scanning Tunneling Microscopy Study. Langmuir 2005, 21, 6915–6928. 10.1021/la0507737. 16008404

[ref71] Tahara K.; Furukawa S.; Uji-i H.; Uchino T.; Ichikawa T.; Zhang J.; Mamdouh W.; Sonoda M.; De Schryver F. C.; De Feyter S.; Tobe Y. Two-Dimensional Porous Molecular Networks of Dehydrobenzo[12]annulene Derivatives *via* Alkyl Chain Interdigitation. J. Am. Chem. Soc. 2006, 128, 16613–16625. 10.1021/ja0655441. 17177410

[ref72] Ha E. H.; Jo M. Y.; Park J.; Kang Y. C.; Yoo S.; Kim J. H. Inverted Type Polymer Solar Cells with Self Assembled Monolayer Treated ZnO. J. Phys. Chem. C 2013, 117, 2646–2652. 10.1021/jp311148d.

[ref73] Kondo T.; Takakusagi S.; Uosaki K. Stability of Underpotentially Deposited Ag Layers on a Au(111) Surface Studied by Surface X-Ray Scattering. Electrochem. Commun. 2009, 11, 804–807. 10.1016/j.elecom.2009.01.036.

[ref74] Horcas I.; Fernandez R.; Gomez-Rodriguez J. M.; Colchero J.; Gomez-Herrero J.; Baro A. M. WSXM: A Software for Scanning Probe Microscopy and a Tool for Nanotechnology. Rev. Sci. Instrum. 2007, 78, 013705 10.1063/1.2432410. 17503926

[ref75] Nefedov A.; Wöll C. Advanced Applications of NEXAFS Spectroscopy for Functionalized Surfaces. In Surface Science Techniques; Bracco G., Holst B., Eds.; Springer Series in Surface Science; Springer-Verlag: Berlin, 2013; Vol. 51, pp 277–306.

[ref76] Moulder J. F.; Stickle W. E.; Sobol P. E.; Bomben K. D. Handbook of X-Ray Photoelectron Spectroscopy; Chastian J., Ed.; Perkin-Elmer Corp.: Eden Prairie, MN, 1992.

[ref77] Stöhr J. NEXAFS Spectroscopy; Springer Series in Surface Sciences; Springer: Berlin, 1992; pp 276–291.

[ref78] Batson P. E. Carbon-1s Near-Edge-Absorption Fine-Structure in Graphite. Phys. Rev. B: Condens. Matter Mater. Phys. 1993, 48, 2608–2610. 10.1103/PhysRevB.48.2608. 10008656

[ref79] Blum V.; Gehrke R.; Hanke F.; Havu P.; Havu V.; Ren X.; Reuter K.; Scheffler M. *Ab Initio* Molecular Simulations with Numeric Atom-Centered Orbitals. Comput. Phys. Commun. 2009, 180, 2175–2196. 10.1016/j.cpc.2009.06.022.

[ref80] Marek A.; Blum V.; Johanni R.; Havu V.; Lang B.; Auckenthaler T.; Heinecke A.; Bungartz H.-J.; Lederer H. The ELPA Library: Scalable Parallel Eigenvalue Solutions for Electronic Structure Theory and Computational Science. J. Phys.: Condens. Matter 2014, 26, 213201 10.1088/0953-8984/26/21/213201. 24786764

[ref81] Auckenthaler T.; Blum V.; Bungartz H.-J.; Huckle T.; Johanni R.; Kraemer L.; Lang B.; Lederer H.; Willems P. R. Parallel Solution of Partial Symmetric Eigenvalue Problems from Electronic Structure Calculations. Parallel Computing 2011, 37, 783–794. 10.1016/j.parco.2011.05.002.

[ref82] Havu V.; Blum V.; Havu P.; Scheffler M. Efficient O (N) Integration for All-Electron Electronic Structure Calculation Using Numeric Basis Functions. J. Comput. Phys. 2009, 228, 8367–8379. 10.1016/j.jcp.2009.08.008.

[ref83] Perdew J. P.; Burke K.; Ernzerhof M. Generalized Gradient Approximation Made Simple. Phys. Rev. Lett. 1996, 77, 3865–3868. 10.1103/PhysRevLett.77.3865. 10062328

[ref84] Ruiz V. G.; Liu W.; Zojer E.; Scheffler M.; Tkatchenko A. Density-Functional Theory with Screened van der Waals Interactions for the Modeling of Hybrid Inorganic-Organic Systems. Phys. Rev. Lett. 2012, 108, 146103 10.1103/PhysRevLett.108.146103. 22540809

[ref85] Tkatchenko A.; Scheffler M. Accurate Molecular van der Waals Interactions from Ground-State Electron Density and Free-Atom Reference Data. Phys. Rev. Lett. 2009, 102, 073005 10.1103/PhysRevLett.102.073005. 19257665

[ref86] Neugebauer J.; Scheffler M. Adsorbate-Substrate and Adsorbate-Adsorbate Interactions of Na and K Adlayers on Al(111). Phys. Rev. B: Condens. Matter Mater. Phys. 1992, 46, 16067–16080. 10.1103/PhysRevB.46.16067. 10003746

[ref87] Tersoff J.; Hamann D. R. Theory of the Scanning Tunneling Microscope. Phys. Rev. B: Condens. Matter Mater. Phys. 1985, 31, 805–813. 10.1103/PhysRevB.31.805. 9935822

[ref88] Heimel G.; Romaner L.; Brédas J. L.; Zojer E. Organic/Metal Interfaces in Self-Assembled Monolayers of Conjugated Thiols: A First-Principles Benchmark Study. Surf. Sci. 2006, 600, 4548–4562. 10.1016/j.susc.2006.07.023.

[ref89] Stukowski A. Visualization and Analysis of Atomistic Simulation Data with OVITO–the Open Visualization Tool. Modell. Simul. Mater. Sci. Eng. 2010, 18, 015012 10.1088/0965-0393/18/1/015012.

[ref90] Larsen A. H.; Mortensen J. J.; Blomqvist J.; Castelli I. E.; Christensen R.; Dułak M.; Friis J.; Groves M. N.; Hammer B.; Hargus C.; Hermes E. D.; Jennings P. C.; Jensen P. B.; Kermode J.; Kitchin J. R.; Kolsbjerg E. L.; Kubal J.; Kaasbjerg K.; Lysgaard S.; Maronsson J. B.; et al. The Atomic Simulation Environment - A Python Library for Working with Atoms. J. Phys.: Condens. Matter 2017, 29, 273002. 28323250 10.1088/1361-648X/aa680e

[ref91] Kokalj A. XCrySDen - A New Program for Displaying Crystalline Structures and Electron Densities. J. Mol. Graphics Modell. 1999, 17, 176–179. 10.1016/S1093-3263(99)00028-5. 10736774

[ref92] Hunter J. D. Matplotlib: A 2D Graphics Environment. Comput. Sci. Eng. 2007, 9, 90–95. 10.1109/MCSE.2007.55.

