# Supplementary material for: Porous Honeycomb Self-Assembled Monolayers: Tripodal Adsorption and Hidden Chirality of Carboxylate Anchored Triptycenes on Ag
Source: ACS Nano. 2021 Jun 14;15(7):11168–79. doi: 10.1021/acsnano.1c03626 (PMC8320238; doi:10.1021/acsnano.1c03626)
Supplement: Supplementary file 1 — nn1c03626_si_001.pdf [file nn1c03626_si_001.pdf]

Supporting Information

Porous Honeycomb Self-Assembled Monolayers: Tripodal Adsorption and Hidden Chirality of Carboxylate Anchored Triptycenes on Ag

Saunak Das,[1] Giulia Nascimbeni,[2] Rodrigo Ortiz de la Morena,[3] Fumitaka Ishiwari,[4] Yoshiaki Shoji,[4] Takanori Fukushima,*[,4] Manfred Buck,*[,3] Egbert Zojer,*[,2] and Michael Zharnikov*[,1]

[1]*Angewandte Physikalische Chemie, Universität Heidelberg, Im Neuenheimer Feld 253, D-69120 Heidelberg, Germany*

[2] *Institute of Solid State Physics, NAWI Graz, Graz University of Technology, Petersgasse 16, 8010 Graz, Austria*

[3] *EaStCHEM School of Chemistry, University of St Andrews, North Haugh, St Andrews KY16 9ST, UK*

[4] *Laboratory for Chemistry and Life Science, Institute of Innovative Research, Tokyo Institute of Technology, 4259 Nagatsuta, Midori-ku, Yokohama 226-8503, Japan*

1. NEXAFS Spectroscopy Data

The C K-edge and O K-edge spectra of the Trip-CA SAM UPD-Ag/Au/mica are shown in Figures S1 and S2, respectively. The spectra corresponding to the magic incidence angle (~55°) are most representative of the electronic structure of the SAMs since they are not affected by molecular orientation.[1] In contrast, the variation of the resonance intensity with X-ray incidence angle, emphasized additionally by the difference spectra, is characteristic of the molecular orientation in the monolayer.[1]

The C K-edge spectra in Figure S1 show the expected absorption features of the Trip-CA molecules. These spectra are dominated by a pronounced π_1* resonance of phenyl rings at ~285.1 eV (π_{ph}*),[1,2] typical also of aromatic SAM containing these moieties,[3,4] including triptycene-based ones.[5,6] Another prominent feature is the characteristic π_1* resonance of CA/carboxylate at ~288.2 eV (π^*_{COO}).[3,7] These features are accompanied by a variety of the less prominent π* and σ* resonances, associated mostly with the triptycene framework.[5,6] Significantly, the intensity of both π^*_{ph} and π^*_{COO} resonances decreases significantly at going

from the normal (90°) to grazing (20°) incidence, which is additionally emphasized by the respective positive peaks in the difference spectrum. Considering that these orbitals are directed perpendicular to the planes of the phenyl and carboxylate groups in the Trip-CA SAM, an upright orientation of triptycene moieties can be assumed, which agrees well with the carboxylate-type bonding of all three anchoring groups of Trip-CA molecules, as follows from the XPS data (see above).

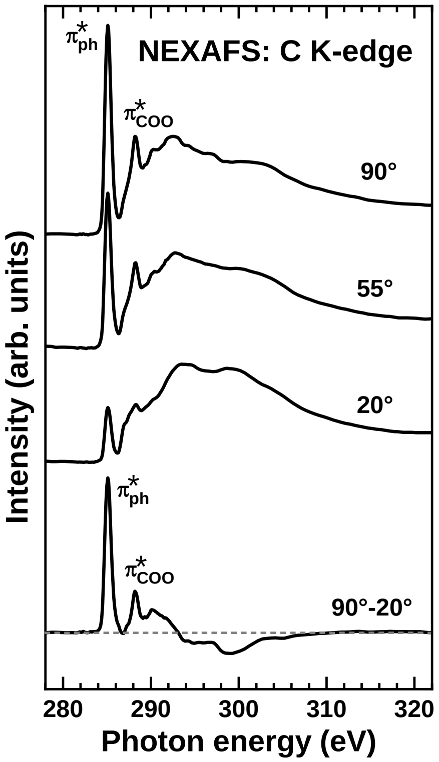

Figure S1. C K-edge NEXAFS spectra of the Trip-CA SAM UPD-Ag/Au/mica (P-phase) acquired at the different X-ray incidence angles, along with the difference between the spectra collected under the normal (90°) and grazing (20°) incidence (bottom curve). Individual absorption resonances are marked (see text for details). The horizontal dashed line corresponds to zero.

A quantitative evaluation of the dependence of the π^*_{ph} intensity on the X-ray incidence angle, performed within the standard theoretical framework for a vector-type orbital,[1] adapted specifically to the triptycene case,[5,6] gives an average tilt angle of ~9° with respect to the surface normal for the axis of the Trip-CA, which is nearly perpendicular to the substrate. This value is quite close to the analogous parameter for the SAMs of 1,8,13-trimercaptomethyltriptycene (~7.5°) also exhibiting a tripodal bonding to the substrate, mediated by the thiolate anchoring groups connected to the triptycene framework over the methylene linker.[6] The deviation from

the fully in-plane orientation of the Trip-CA molecules in the SAM with respect to the substrate could be, on the one hand, explained by a possible corrugation of the specific anchoring sites of individual carboxylate groups and on the other hand, by the presence of disordered amorphous phase (see section 3.1) as well as by occurrence of defects, such as domain boundaries or step edges, distorting the arrangement of the Trip-CA molecules within the P-phase.

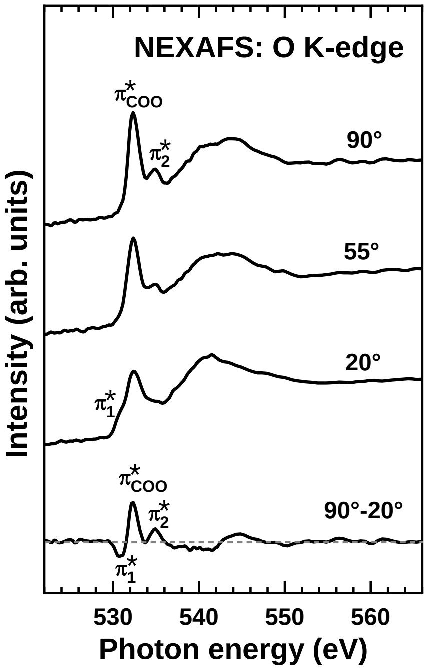

Figure S2. O K-edge NEXAFS spectra of the Trip-CA SAM UPD-Ag/Au/mica (P-phase) acquired at the different X-ray incidence angles, along with the difference between the spectra collected under the normal (90°) and grazing (20°) incidence (bottom curve). Individual absorption resonances are marked (see text for details). The horizontal dashed line corresponds to zero.

The π^*_{COO} resonance is also observed in the O K-edge NEXAFS spectra of the Trip-CA SAM (Figure S2), appearing at a typical PE of ~532.3 eV (see, *e.g.*, refs [3] and [7]) and representing the dominant feature there. This feature is accompanied by an additional π^* resonance at higher PE (534.9 eV; π_2^*), typical of COOH/COO^-,[1,8] and by a variety of σ^* resonances at higher PEs. In addition, there is a weak π_1^* resonance at 530.8 eV, appearing as a shoulder of the π^*_{COO} feature in the 20° spectrum. This peak has been frequently observed in different CA SAMs on UPD-Ag/Au/mica[3,9] but not rationalized so far.

In agreement with the C K-edge spectra and the proposed molecular orientation, the intensities of the π^*_{COO} and π_2^* resonances in Figure S2 decrease significantly at going from the normal to grazing incidence, corresponding to the positive peaks in the difference spectrum. In contrast, the π_1^* resonance exhibits an opposite behavior, emphasized by the negative peak in the difference spectrum. This suggests a strong hybridization of a part of the π^* orbitals of the carboxylate groups with the electronic states of the substrate, resulting in their reorientation and lowering of energy.

2. Additional Computational Data

2.1. Details on the Employed Basis Functions

The basis functions employed in the FHI-aims simulations have the format

$$\Phi(r) = \frac{u(r)}{r} * Y_{lm}(\Theta, \Phi)$$

in spherical coordinates (r, Θ, Φ). They are defined relative to a given atomic center and are provided together with the FHI-aims code. Default settings for four different levels of accuracy are contained in the species_defaults subdirectory. For the production calculations, "tight" settings (*i.e.*, the second-highest setting) have been used. They comprise the functions described in Table S1.

Table S1. Basis functions that have been used for the calculations performed with FHI-aims. The abbreviations read as follows (as described in FHI-aims: A User's Guide: February 10, 2018): X(nl, z), where X describes the type of basis function where H stands for hydrogen-like functions and ionic for a free-ion like radial function. The parameter n stands for the main/radial quantum number, l denotes the angular momentum quantum number (s, p, d, f, …), and z denotes an effective nuclear charge, which scales the radial function in the defining Coulomb potential for the hydrogen-like function. In the case of free-ion like radial functions, z specifies the onset radius of the confining potential. If auto is specified instead of a numerical value, the default onset is used.

	**H**	**C**	**O**	**Au**	**Ag**
**Minimal**	valence(1s, 1.0)	valence(2s, 2.0)<br>valence(2p, 2.0)	valence(2s, 2.0)<br>valence(2p, 4.0)	valence(6s, 1.0)<br>valence(5p, 6.0)<br>valence(5d,10.0)<br>valence(4f,14.0)	valence(5s, 1.0)<br>valence(4p, 6.0)<br>valence(5d,10.0)
**First tier**	H(2s, 2.1)<br>H(2p, 3.5)	H(2p, 1.7)<br>H(3d, 6)<br>H(2s, 4.9)	H(2p, 1.8)<br>H(3d, 7.6)<br>H(3s, 6.4)	ionic(6p, auto)<br>H(4f, 7.4)<br>ionic(6s, auto)<br>H(5g, 10.0)<br>H(6h, 12.8)<br>H(3d, 2.5)	ionic(5p, auto)<br>H(4f, 7.6)<br>H(3s, 2.6)<br>H(5g, 9.8)<br>H(4d, 8.4)
**Second tier**	H(1s, 0.85)<br>H(2p, 3.7)<br>H(2s, 1.2)<br>H(3d, 7.0)	H(4f, 9.8)<br>H(3p, 5.2)<br>H(3s, 4.3)<br>H(5g, 14.4)<br>H(3d, 6.2)	H(4f, 11.6)<br>H(3p, 6.2)<br>H(3d, 5.6)<br>H(5g, 17.6)<br>H(1s, 0.75)		

For the additional numerical settings, like the integration grids and cutoff potentials, the default settings for "tight" basis functions are used (as described in FHI-aims: A User's Guide: February 10, 2018). Only the settings for the cutoff potential for the Ag basis functions are modified to more accurately describe the electron cloud spilling out of the metal surface (the onset radius of the cutoff potential is increased from 4.0 Å to 4.6 Å and the radial width of the cutoff potential is increased from 2.0 Å to 2.6 Å)

2.2. Considered Starting Structures and Calculated Optimized Configurations for the Porous Phase of Trip-CA on Ag/Au in the Presence of Adatoms

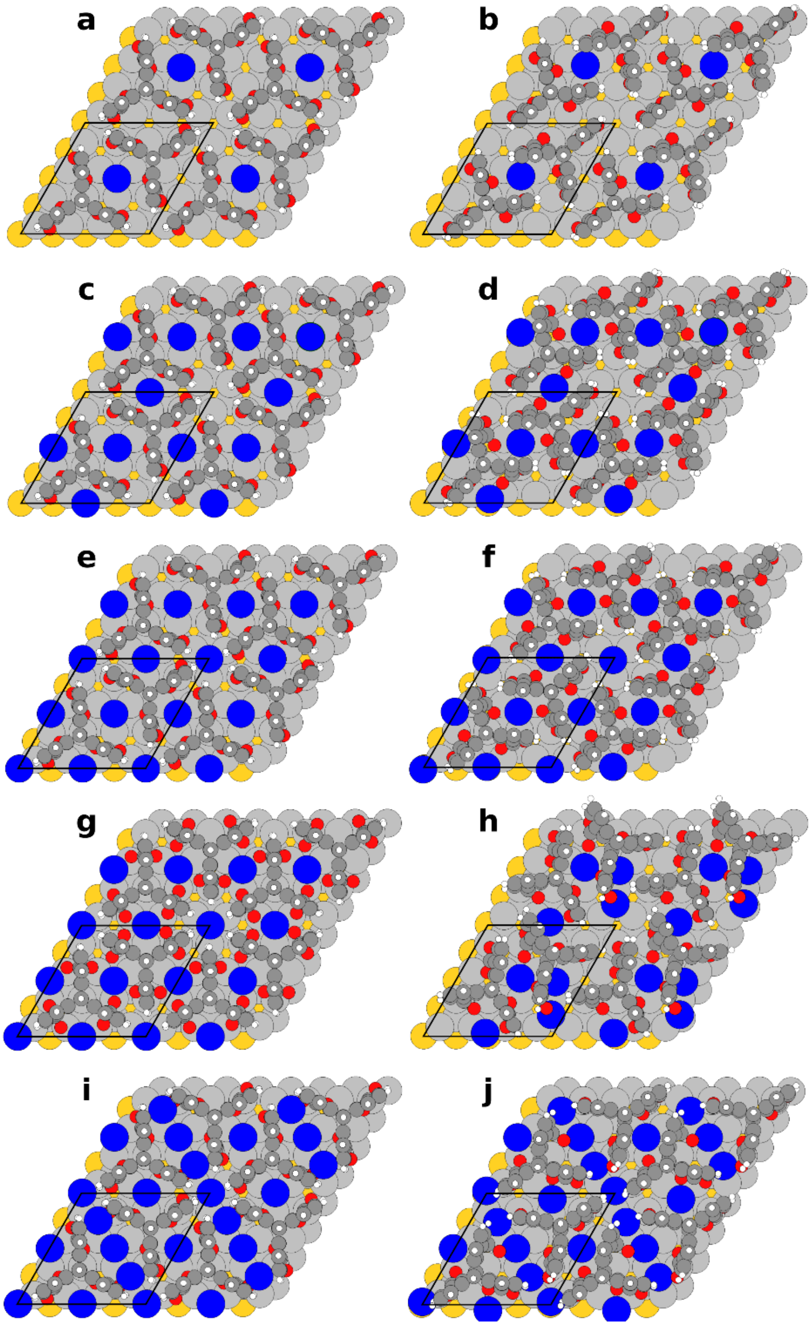

Figure S3. Reconstruction motifs tested to investigate the impact of adatoms. Left panels: starting geometries; right panels: optimized geometries. Au atoms are depicted in dark yellow, Ag atoms in light grey, Ag adatoms in blue, O atoms in red, C atoms in dark grey, and H atoms in white. The unit cells are indicated by the black lines.

2.3. Constant-Current STM Images at Different Heights Above the Adsorbate Layer

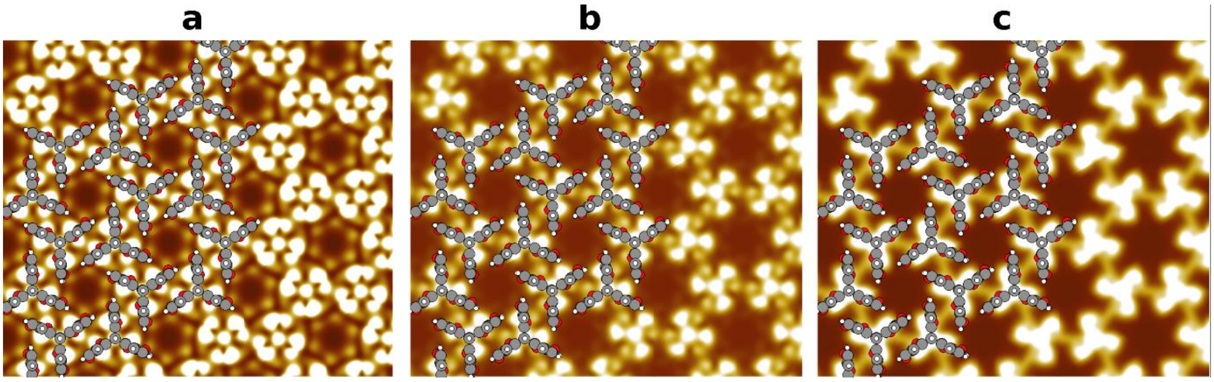

Figure S4. Simulated constant height STM images integrating over all states between the energy just below the HOMO derived feature and the Fermi level (for details see main manuscript). The panels show the situation 0.5 (a), 1.8 (b), and 2.2 (c) Å above the position of the center of the highest atom of the assembled triptycene molecules.

References

(1) Stöhr, J. *NEXAFS Spectroscopy*; *Springer Series in Surface Sciences*; Springer: Berlin, 2003.

(2) Horsley, J.; Stöhr, J.; Hitchcock, A. P.; Newbury, D. C.; Johnson, A. L.; Sette, F. Resonances in the K Shell Excitation Spectra of Benzene and Pyridine: Gas Phase, Solid, and Chemisorbed States. *J. Chem. Phys.* **1985**, *83*, 6099–6107.

(3) Aitchison, H.; Lu, H.; Hogan, S. W. L.; Fruchtl, H.; Cebula, I.; Zharnikov, M.; Buck, M. Self-Assembled Monolayers of Oligophenylenecarboxylic Acids on Silver Formed at the Liquid-Solid Interface. *Langmuir* **2016**, *32*, 9397−9409.

(4) Frey, S.; Stadler, V.; Heister, K.; Eck, W.; Zharnikov, M.; Grunze, M.; Zeysing, B.; Terfort, A. Structure of Thioaromatic Self-Assembled Monolayers on Gold and Silver. *Langmuir* **2001**, *17*, 2408–2415.

(5) Liu, J.; Kind, M.; Schüpbach, B.; Käfer, D.; Winkler, S.; Zhang, W.; Terfort, A.; Wöll, C. Triptycene-Terminated Thiolate and Selenolate Monolayers on Au(111). *Beilstein J. Nanotechnol.* **2017**, *8*, 892–905.

(6) Ishiwari, F.; Nascimbeni, G.; Sauter, E.; Tago, H.; Shoji, Y.; Fujii, S.; Kiguchi, M.; Tada, T.; Zharnikov, M.; Zojer, E.; Fukushima, T. Triptycene Tripods for the Formation of Highly Uniform and Densely Packed Self-Assembled Monolayers with Controlled Molecular Orientation. *J. Am. Chem. Soc.* **2019**, *141*, 5995−6005.

(8) Urquhart, S. G.; Ade, H. Trends in the Carbonyl Core (C 1S, O 1S) $\rightarrow \sigma^*_{C=O}$ Transition in the Near-Edge X-Ray Absorption Fine Structure Spectra of Organic Molecules. *J. Phys. Chem. B* **2002**, *106*, 8531-8538.

(9) Cebula, I.; Lu, H.; Zharnikov, M.; Buck, M. Monolayers of Trimesic and Isophthalic Acid on Cu and Ag: The Influence of Coordination Strength on Adsorption Geometry. *Chem. Sci.* **2013**, *4*, 4455–4464.
